# SENP5 promotes homologous recombination-mediated DNA damage repair in colorectal cancer cells through H2AZ deSUMOylation

**DOI:** 10.1186/s13046-023-02789-9

**Published:** 2023-09-08

**Authors:** Tingting Liu, Hang Wang, Yuanyuan Chen, Zhijie Wan, Zhipeng Du, Hui Shen, Yue Yu, Shengzhe Ma, Ying Xu, Zhuqing Li, Nanxi Yu, Fangxiao Zhang, Kun Cao, Jianming Cai, Wei Zhang, Fu Gao, Yanyong Yang

**Affiliations:** 1 Department of Radiation Medicine, Faculty of Naval Medicine, Naval Medical University, Shanghai, China; 2 School of Public Health and Management, Wenzhou Medical University, Wenzhou, Zhejiang China; 3 Department of Colorectal Surgery, Changhai Hospital, Naval Medical University, Shanghai, China

**Keywords:** Cancer resistance, SENP5, deSUMOylation, DNA damage repair

## Abstract

**Background:**

Neoadjuvant radiotherapy has been used as the standard treatment of colorectal cancer (CRC). However, radiotherapy resistance often results in treatment failure. To identify radioresistant genes will provide novel targets for combined treatments and prognostic markers.

**Methods:**

Through high content screening and tissue array from CRC patients who are resistant or sensitive to radiotherapy, we identified a potent resistant gene SUMO specific peptidase 5 (SENP5). Then, the effect of SENP5 on radiosensitivity was investigated by CCK8, clone formation, comet assay, immunofluorescence and flow cytometric analysis of apoptosis and cell cycle to investigate the effect of SENP5 on radiosensitivity. SUMO-proteomic mass spectrometry combined with co-immunoprecipitation assay were used to identify the targets of SENP5. Patient-derived organoids (PDO) and xenograft (PDX) models were used to explore the possibility of clinical application.

**Results:**

We identified SENP5 as a potent radioresistant gene through high content screening and CRC patients tissue array analysis. Patients with high SENP5 expression showed increased resistance to radiotherapy. In vitro and in vivo experiments demonstrated that SENP5 knockdown significantly increased radiosensitivity in CRC cells. SENP5 was further demonstrated essential for efficient DNA damage repair in homologous recombination (HR) dependent manner. Through SUMO mass spectrometry analysis, we characterized H2AZ as a deSUMOylation substrate of SENP5, and depicted the SUMOylation balance of H2AZ in HR repair and cancer resistance. By using PDO and PDX models, we found targeting SENP5 significantly increased the therapeutic efficacy of radiotherapy.

**Conclusion:**

Our findings revealed novel role of SENP5 in HR mediated DNA damage repair and cancer resistance, which could be applied as potent prognostic marker and intervention target for cancer radiotherapy.

**Supplementary Information:**

The online version contains supplementary material available at 10.1186/s13046-023-02789-9.

## Background

Colorectal cancer (CRC) is currently the third most common cancer and the second most leading cause of cancer-related mortality worldwide [1]. Although significant advances have been obtained in molecular and immunotherapeutic approaches, the prognosis and treatment for the advanced disease remain poor [2, 3]. In recent years, neoadjuvant radiotherapy has been used as the standard treatment of CRC, especially local advanced rectal cancer [4, 5]. However, original or gradually adaptive radiotherapy resistance often results in treatment failure in clinical practice [6, 7]. Among all aspects in radiosensitivity, abnormally enhanced DNA damage repair represents a critical factor leading to cancer resistance [8, 9]. Mutations in DNA damage repair genes, including homologous recombination repair and mismatch repair, are also common in the pathogenesis of colorectal cancer [10, 11]. To uncover novel mechanism in DNA damage repair for CRC to resist radiotherapy will provide great opportunity for overcoming cancer resistance.

The most severe type of DNA damage induced by ionizing radiation (IR) is double strand breaks (DSBs), which is an important upstreaming molecular events that can trigger a series of cellular DNA damage responses (DDRs) and lead to a variety of cellular responses such as cell cycle arrest, apoptosis, autophagy, and senescence [8]. DSBs are often repaired through two groups of highly organized biological processes including nonhomologous end joining (NHEJ) and homologous recombination (HR) [12]. For decades, numerous repair factors involved in these two pathways were identified as rapid mobilization and recruitment through post-translational modifications (PTMs) shortly after DNA damages happen. For instance, the three key PI3K-related kinase (PIKK) family members, DNA-PK, ATM and ATR phosphorylates series of substrates which activates cell cycle arrest as well as both NHEJ and HR repair [13, 14]. Ubiquitination system, such as RNF8/168, USP family, also regulates the degradation or activation of the downstream factors [15]. Besides, critical roles of histone acetylation, methylation, ubiquitination in DNA damage repair were also recognized [16]. Different types of modifications alter the charge state, hydrophobicity, conformation and stability of proteins, and ultimately affect their functions.

Recently, SUMOylation have received increasing attention for their crucial roles in a variety of biological processes such as DNA damage repair and programmed cell death [17]. Basically, SUMOylation is a reversible enzymatic cascade reaction in which E1 activating enzyme, E2 binding enzyme and E3 ligase act synergistically to bind SUMO proteins to specific lysine sites, whereas de-SUMOylation is the removal of SUMO molecules from the target protein by the family of Sentrin-specific proteases (SENPs) [17, 18]. In the mammalian cell, the SENP family is composed of seven members, SENP1-3 and SENP5-8. SUMOylation balances of several DNA damage repair proteins, including MDC1, BRCA1, ATRIP, were also reported [19–21]. Several members of SENPs overexpression positively correlated with clinicopathological features such as cancer aggressiveness, and recurrence [22, 23].

In our recent study, we performed a CRIPSR Cas9 screening combined with irradiation and identified SENP5 as a potent target related to radiation resistance. We further analyzed SENP5 expression in CRC and found important clinical relevance with radiotherapy. Even SENP5 is predominantly found in the nucleus with potential regulating DNA damage repair, little is known about its role and potential deSUMOylation substrates in DNA damage repair. In our present study, we introduced our findings of SENP5 in radiotherapy resistance and revealed its novel mechanism in HR-mediated DNA damage repair through deSUMOylation of H2AZ. SENP5 was also validated as a potent target for cancer therapy in patient-derived preclinical models.

## Methods

### Cell lines

The HIEC, NCM-460, HCT116, LOVO, HT29, CACO-2, RKO, SW620, SW480 and HEK-293T cell lines were from the American Type Culture Collection (ATCC) and were cultured under conditions specified by the manufacturer. HCT116 cells were cultured in McCoy 5a medium with 10% fetal bovine serum. HIEC and NCM-460 cells were cultured in Opti-MEM reduced serum medium with 4% fetal bovine serum, 20 mM hepes, 10 mM glutamax (#35,050, Gibco, USA) and 10 ng/mL epidermal growth factor. LOVO cells were cultured in RPMI 1640 medium with 10% fetal bovine serum. RKO were cultured in eagle’s minimum essential medium with 10% fetal bovine serum. HCT116, HT29, CACO-2, HEK-293T, SW620 and SW480 were cultured in Dulbecco’s modified eagle medium with 10% fetal bovine serum. Short tandem repeat profiling (for cell line authentication) and mycoplasma tests were done before experiment.

### Plasmids, lentivirus package and infection

Lentiviral shRNA vectors for human cells included non-targeting control and shSENP5 #1/2/3 were produced by Hanbio Tech (Shanghai, China). The short hairpin RNA (shRNA) sequences were as follows: shSENP5-1:5’-CACAACCTTCTGACTTTCCCATGAA-3’, shSENP5-2:5’-GGGAGTGTACAGAGCTGATTCATGA-3’, shSENP5-3:5’-CAGTCCCAGACAAAGTTCACTTCTT-3’. The SENP5 wild-type and SENP5 C713L overexpression plasmid were directly synthesized by OBIO Technology (Shanghai, China). The H2AZ wild-type and H2AZ 3KR overexpression plasmids were synthesized by Genomeditech (Shanghai, China). The viral vector and packaging plasmids (psPAX2 and pMD2.G) were co-transfected into HEK-293T cells, and then virus-containing supernatant was collected at 48 and 72 h after co-transfection. Viral supernatant was filtered through 0.45 μm filters and infect cells using 10 g/mL Polybrene. Stable cell lines were selected on medium containing 1 μg/mL puromycin or 2 μg/mL blasticidin.

### RNA interference assay

The cells were seeded 24 h prior to transfection to yield a density of 70–80% confluence at the time of transfection. Liposomal cocktails with siRNA (20 nM final) were generated with Lipofectamine 3000 (#L3000008, Invitrogen, USA) in Opti-MEM following manufacturer’s recommendations. Fresh culture medium was changed 24 h after transfection. Transfected cells were incubated for 48 h prior to use. The siRNAs against human H2AZ were purchased from Hanbio Tech (HH20221201SHQJL, Shanghai, China).

### RNA extraction and real-time PCR

RNA was extracted using total RNA Extraction Kit (R2000, Solarbio, China) following manufacturer’s recommendations. RNA quality and quantity were determined using Nanodrop ND-1000 Spectrophotometer (Thermo Fisher Scientific, USA), and stored at − 80°C. Reverse transcription assay was performed using the Script Reverse Transcription Supermix Kit (#RR047A, Takara, Japan) according to the manufacturer’s instructions. Real-time PCR was performed using Power SYBR Green PCR master mix (#RR430A, Takara, Japan). For quantification of gene expression, the 2^−ΔΔCT^ method was used and the data was normalized to an endogenous control (GAPDH). The sequence information for each primer used for gene expression analysis is as follows: SENP5 Forward: 5’-GGGAAGGCCAGTTACTTGGAA-3’; SENP5 Reverse: 5’- CAAAGGGGTTCATCCTTGATCC-3’.

GAPDH Forward: 5’-CAGGAGGCATTGCTGATGAT-3’; GAPDH Reverse: 5’-GAAGGCTGGGGCTCATTT-3’.

### CCK-8 assay

Cells were seeded in 96-well plates with 3 × 10^3^ per well. At 24, 48 and 72 h after radiation, cell counting kit-8 reagent (#CK04, Dojindo, Japan) was added and the cells were incubated at 37 °C for 2 h. Absorbance was measured in a microplate reader (Beckman Coulter, USA) at 450 nm.

### Colony formation assay

Radiosensitivity of tumor cells was assessed using the colony formation assay. Before irradiation, a single-cell suspension of exponentially growing cells was plated on six-well dishes. Subsequently, cells were irradiated with the indicated dose and allowed to grow for additional 10–14 days. The resulting colonies were fixed with paraformaldehyde (4%) for 15 min and stained with crystal violet (5%) for 15 min. After washing three times with PBS, the colonies of more than 50 cells were counted in each group. Experiments were repeated in triplicate. Data from experimental groups were normalized to their respective control groups.

### Cell-cycle analysis

To analyze the effects of treatment on cell cycle distribution, we seeded 10^6^ of cells into 6-well plates. The next day, the cells were attached to the plate and then treated with irradiation. Next, cells were trypsinized at different time points(0, 6, 12, 24 h), washed with phosphate-buffered saline (PBS), and fixed in ice-cold 70% ethanol overnight. After fixation, cells were washed with PBS and stained with a propidium iodide staining solution (50 ug/mL propidium iodide and 100 ng/mL RNase A) at 37 °C for at least 20 min. Data were collected with Flow Cytometer system (Beckman Coulter, USA) and cell-cycle analysis was performed with FlowJo software.

### Apoptosis assay

Apoptotic cells were detected using the Annexin V-APC/PI apoptosis kit (#AP107, Lianke Bioscience, China) according to the manufacturer’s protocol. Briefly, 2 × 10^5^ cells and supernatant were collected by using non-EDTA trypsin. After washed with PBS, the cell pellets were resuspended with binding buffer. Cells in each group were added with 5ul Annexin V and 5ul PI, and then incubated for 30 min away from light. APC and PI channels were used to detect apoptosis. Samples were analyzed using Flow Cytometer system (Beckman Coulter, USA) and data analyzed with FlowJo Software.

### Western blot assay

Cells were treated as indicated and harvested for protein extraction. Cells were rinsed with PBS, scraped, and lysed in ice-cold extraction buffer (#89,900, Thermo Fisher Scientific, USA) containing protease and phosphatase Inhibitor Cocktail (#78,447, Thermo Fisher Scientific, USA). Lysates were briefly sonicated, clarified, then subjected to SDS-PAGE and transferred to PVDF membranes using a Bio-Rad transfer apparatus. Membranes were blocked with 5% non-fat milk in TBST at room temperature for 1 h, followed by incubation with primary antibody overnight at 4 °C. On the following day, the membranes were washed 3 × 5 min in TBST and incubated with species-specific HRP-conjugated secondary antibodies for 1 h. Next, after 3 × 10 min washes in TBST, membranes were developed using an enhanced chemiluminescence reagent before being exposed to ChemiDoc Imaging System (6000plus, bltlux, China). Molecular weight markers were used to determine the size of proteins. Protein bands were quantified using ImageJ software (Image J software, National Institutes of Health, USA). Antibodies were listed in Additional file S1.

### Co-immunoprecipitation (Co-IP) assay

Cells were harvested at 8 h after 8 Gy irradiation and lysed in IP lysis buffer (50 mM Tris-HCl [pH 7.4], 125 mM NaCl, 1 mM EDTA, 0.1% Triton X-100) with protease and phosphatase inhibitors. Protein concentration was then determined using a protein quantification kit (#P0012S, Beyotime, China) to ensure that equal amounts of total protein were loaded in each group. Lysates were then incubated with protein G agarose beads and IgG antibody of the same species as the IP antibody for 10 h at 4 °C to reduce non-specific binding. The cleared lysates were then incubated with IP antibody overnight at 4 °C. Beads were washed with IP lysis buffer, boiled in 1× SDS gel loading buffer, and subjected to electrophoresis as described above.

### SUMOylation assay

For detection of endogenously SUMOylated H2AZ, cells were left untreated or treated with irradiation for 4 h. Cells were harvested and lysed in the NETN lysis buffer (50 mM Tris–HCl, pH 7.4, 100 mM sodium chloride, 0.4% NP-40, 1 mM EDTA), sonicated, and boiled at 100° C for 5 min. Cell lysates were then centrifuged at 14,000 rpm for 10 min at 4° C, and the resulting supernatants were diluted 1:10 in RIPA dilution buffer (50 mM Tris-HCl pH 8.0, 150 mM NaCl, 1% NP-40, 0.25% Sodium deoxycholate) containing NEM and protease inhibitors. Lysates were subsequently incubated with protein A + G beads combined with 2 ug of rabbit IgG or H2AZ antibody for 4 h at 4 ° C with gentle rocking. The resin was washed three times with NETN buffer. The protein-bound beads were boiled in 1× SDS loading buffer and subjected to Western blotting.

### RNA sequencing

RNA samples were collected from NC and shSENP5 cells. RNA isolation, library construction, and RNA sequencing (RNA-seq) were carried out following standard protocols. The library products were sequenced using a BGISEQ-500. Standard bioinformatics analysis was performed by the Beijing Genomics Institute. For gene expression analysis, the significance of the differential expression genes was defined by the bioinformatics service of BGI according to the combination of the absolute value of |log2FC|≥1 and q value < 0.05. GSEA analysis was performed using the OmicShare tools, a free online platform for data analysis (https://www.omicshare.com/tools).

### Neutral comet assay

A neutral comet assay was conducted using a CometAssay kit (#4250-050-K, Trevigen, USA) following the manufacturer’s instructions. Briefly, untreated cells or cells subjected to IR (8 Gy, 2 h) were digested with trypsin and resuspended in ice-cold PBS at a concentration of 2 × 10^5^ cells/mL. Next, 50μL of cells suspension were mixed with 500 μl preheated comet LMAgarose, immediately placed on the center of object slides, and left for 30 min at 4 °C until a 0.5 mm clear ring appears at edge of CometSlide™ area. Immerse slides in 4 °C Lysis Solution overnight for added sensitivity. Following washing with neutral electrophoresis buffer (100mM tris base,300mM Sodium Acetate, pH 9.0) for 30 min, the samples were subjected to electrophoresis at 21 volts and apply voltage for 45 min at 4 °C. Afterwards, drain excess neutral electrophoresis buffer and gently immerse slides in DNA Precipitation Solution (7.5 M NH4Ac in 95% EtOH) for 30 min and then immerse slides in 70% ethanol for 30 min at room temperature. Slides were dried overnight and stained with SYBR green I (Invitrogen, 10 mM Tris–HCl, pH 7.5, 1 mM EDTA, pH 8.0). Images were obtained using a Zeiss microscope at ×20 magnification. For each group, tail moments of at least 200 cells were measured by using OpenComet (v1.3.1 from https://cometbio.org), and the olive tail movements are shown.

### Immunofluorescence

For immunostaining of SENP5, γH2AX, 53BP1 and RAD51, cells were cultured in chamber slides (#155,411, Thermo Fisher Scientific, USA) overnight before irradiation. The cells were washed with phosphate buffered saline (PBS), fixed with 4% paraformaldehyde, permeabilized with 0.1% Triton X-100 in PBS. Cells were then blocked for 5% goat serum in PBS at room temperature for 2 h, and incubated with the primary antibodies diluted in PBS-BSA at 4 °C overnight. After three washes with PBS, fluorescently labeled secondary antibodies Alexa Fluor 488 goat anti-rabbit IgG (A11008, Invitrogen, USA) and Alexa Fluor cy3 goat anti-mouse IgG (A11005, Invitrogen, USA) in PBST–BSA were added for 1 h. Coverslips were mounted on slides by using anti-fade mounting medium with DAPI (#G1012, Servicebio, China). Immunofluorescence images were acquired on a Zeiss LSM880 confocal microscope or Olympus FV1000 confocal microscope. Slides were imaged at 63× magnification. Zen 2.6 (Zeiss) software was used for confocal image processing.

### HR and NHEJ reporter assay

DNA repair reporter systems were used to determine the HR and NHEJ efficiency. Firstly, we conducted Hela cells stably expressing NHEJ and HR reporter (#98,895, Addgene, USA) with selected by G418. Then, Hela NHEJ and HR reporter cells were transfected with the SENP5 NC and shRNA respectively. Cells were plated at 3 × 10^5^ cells per well on 12-well plates the day before the transfection. 0.5 μg of I-SceI plasmid and 0.5 ug pCAGGS DRR mCherry Donor EF1a BFP (#98,896) were transfected into the cells with lipo3000. 48 h later, cells were harvested and subjected to flow cytometric analysis to determine the percentage of GFP-positive cell and mCherry -positive cells, which result from NHEJ repair and HR repair induced by DNA DSBs. Means were obtained from 3 independent experiments.

### Xenograft tumor assay

Four-week-old athymic nude mouse strain were used for the xenograft tumor assay in this study. The mice were purchased from Jihui Laboratory and kept in a pathogen-free environment. For cell derived xenograft (CDX) model, the indicated HCT116-NC cells and HCT116-SENP5 KD cells (1 × 10^6^) were subcutaneously injected into the nude mice. A week after tumor cell implantation, we confirmed tumor cell engraftment by measuring tumor size, excluded the outliers, and randomly divided the mice into four treatment groups: (1) HCT116-NC non-irradiation group; (2) HCT116-NC irradiation group; (3) shSENP5 non-irradiation group; and (4) shSENP5 irradiation group. The patient-derived xenograft (PDX) model was purchased from LIDE Biotech (shanghai china). Mice bearing passage 3 PDX model (COPF161282) were randomly divided into 4 groups as described above. When the PDX tumor volume reached 200 mm^3^, each mouse injected with 2 × 10^7^ TU of indicated virus intratumorally for three consecutive days. CDX and PDX tumor bearing mice received a locally 22 Gy exposure, and the residual region of the body shielded with lead, when the tumor size approximately reached 500 mm^3^. Tumor size was measured using a caliper every 2 days, and tumor volume was calculated using the standard formula 0.5×L× W×W, where L is the longest diameter and W is the shortest diameter. Total body weight was measured in two days intervals.

### Immunohistochemistry

Immunohistochemistry was performed as described. Briefly, 5-micron thick formalin-fixed paraffin embedded human tissue sections were stained with the SENP5 (#19529-1-AP, Proteintech, USA), Ki67 (GB111499, Servicebio, China), γ-H2AX(GB111841, Servicebio, China), RAD51 (#ab1837, abcam, US) or p-CHK1 (#ab47318, abcam, US) antibody per manufacturer’s instructions. TUNEL staining was conducted to evaluate apoptosis in resected tissue according to the manufacturer’s instructions (Servicebio, Wuhan, China). Stained slides were digitized using the panoramic slice scanner (3DHISTECH, Hungary) with a 40× objective. Three fields of view per section were used to determine the mean and standard error of the mean of positively staining cells.

### Tissue microarray and Kaplan-Meier survival analysis

Tissue microarray was constructed based on patients’ cancer tissues and tissues adjacent to carcinoma. Two urologic pathologists unaware of the patients’ clinical features and outcomes evaluated these slides. In this study, TRG 0–1 was categorized as the good response group and TRG 3 as the poor response group. IHC assays were conducted to detect SENP5 protein levels in cancer tissues and paracancerous tissues, after which SENP5 levels were independently and semi-quantitatively assessed by using the positive rate (the positive rate = (number of positive cells/total number of cells) × 100%). The cut-off value for SENP5 level was deduced according a receiver operating characteristic curve, and patients were categorized into two groups based on SENP5 level (high or low). Kaplan-Meier survival analysis showed the correlation between SENP5 expression level and overall survival of patients.

### Patient-derived organoids and treatment

In this study, PDO model was established using tumor tissues of colorectal cancer patients in the colorectal surgery department of Shanghai Changhai Hospital. Briefly, freshly removed tumor tissue was cut into approximately 5 mm^3^ and incubated in a digestive buffer (#12,634,010, Gibco, USA) at 37 °C for 1 h on an orbital shaker. The culture was allowed to stand for 3 min and the supernatant was transferred to a new centrifuge tube and centrifuged at 2500 rpm for 1 min. Discard the supernatant, add erythrocyte lysis buffer to dissolve the precipitate, shake for 4 min at room temperature, and centrifuge at 2500 rpm for 1 min. After full resuspension of the pellet with matrix gel (#356,231, Corning, USA), add 30 μL of Matrigel resuspension to each well of a 48-well plate. After complete gelation, 500 μL of colorectal cancer-like organic medium (K2103-CR, Biogenous, China) was added to each well. The medium was changed every 2–3 days, and the organoids were passaged every 5–7 days. The NC and knockdown lentivirus of SENP5 were added separately after the second day of organoid passaging. After culturing for 48 h, organoids were irradiated with 8 Gy γ-rays. Then, images were taken with a light microscope. The organoids area was evaluated with Image J (Image J software, National Institutes of Health, USA).

### SUMO mass spectrometry

Detailed methods about the proteomic analysis were described in Additional file 2.

### Statistics

Each experiment was independently repeated three times and the representative data are shown. Statistical analyses were performed using GraphPad Prism software (version 8.0). All the values are presented as mean ± SD of three biologically independent samples. Statistical analyses were performed using an unpaired two-tailed Student’s t test or a two-way analysis of variance (ANOVA) when comparing at least three groups. P value < 0.05 was considered statistically significant. Error bars, p value, and sample sizes are indicated in figure legends.

## Results

### SENP5 is correlated with radiotherapy resistance in clinical patients and colorectal cancer cell lines

**Fig. 1:**
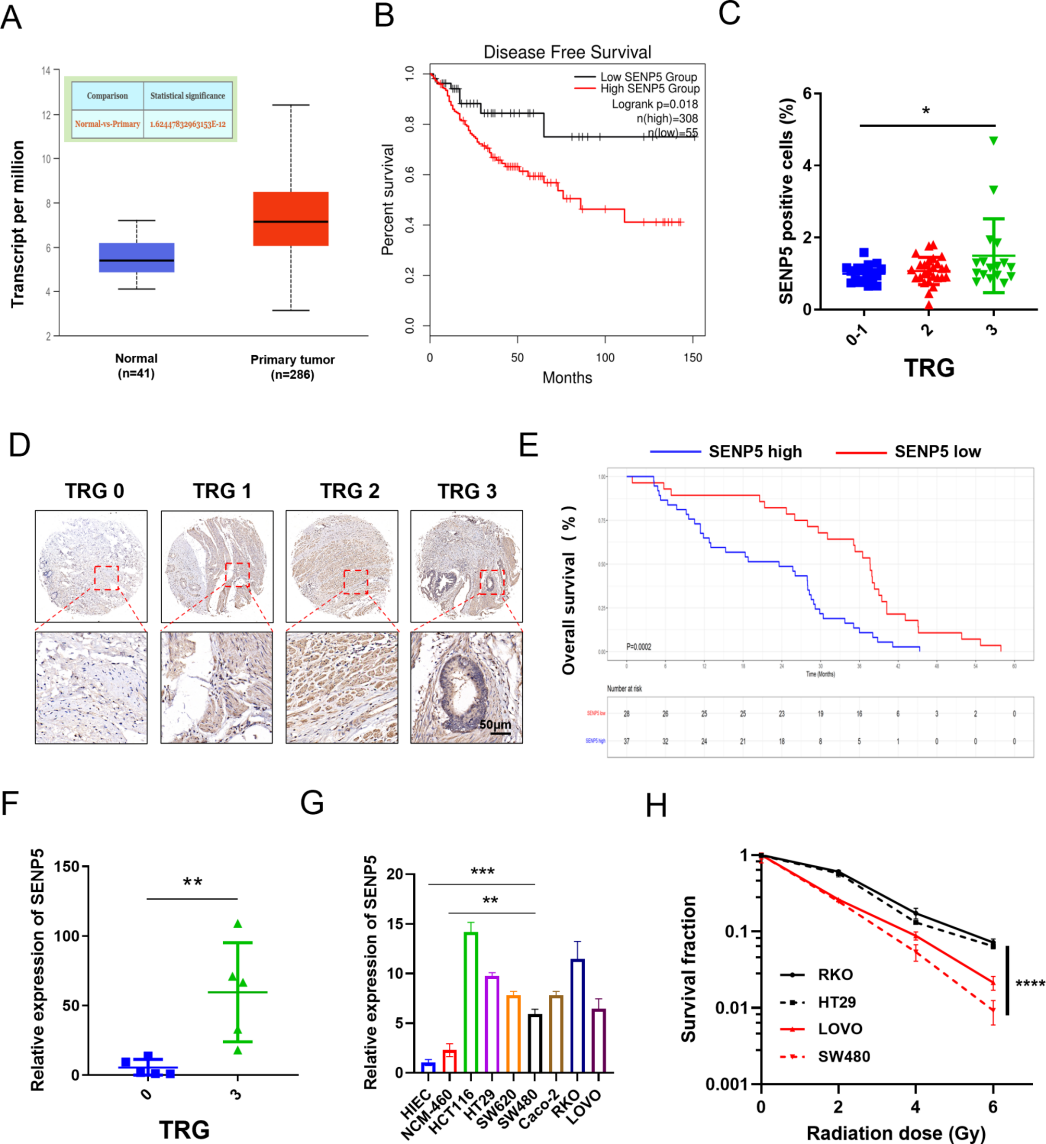
SENP5 was correlated radioresistance in colorectal cancer. **A** Expression of SENP5 in colorectal cancer tissues and normal tissues of CRC patients. These data were acquired from UALCAN (http://ualcan.path.uab.edu) **B** Kaplan-Meier survival analysis SENP5 high expression and low expression in Colorectal cancer (CRC) patients derived from TCGA database (log-rank p = 0.0018). These data were acquired from GEPIA2 (http://gepia2.cancer-pku.cn). **C-D** IHC staining and quantitative analysis of SENP5 in LARC cancer tissues with different radiosensitivity based on tumor regression grades (Radiosensitive, TRG = 0–1, Radioresistant, TRG = 3). *P < 0.05 Vs the TRG0-1 group. **E** Kaplan-Meier survival analysis of SENP5 in LARC patients received radiotherapy. **F** SENP5 mRNA expression in radiosensitive and radioresistant patients measured by RT-PCR assay. **G** The mRNA expressions of SENP5 in normal intestine and CRC cell lines. **H** Colony formation analysis of celluar radiosensitivity in CRC cells with high Vs low SENP5 expressions. ****P < 0.0001 Vs survival in SW480 cells

To screen potent targets for radioresistant genes, we previously performed a CRISPR Cas9 negative screening and identified 200 genes essential for cell survival after irradiation (data not shown). We further analyzed the top 100 genes in CRC using the information derived from the TCGA database. Ten genes were selected based on the following criteria: upregulated in tumor tissues; correlated with poor outcomes (Fig. S1A). The results showed that SENP5 knockdown results in the significant inhibition efficacy in cell proliferation through high content screening in HCT116 cells (Fig. S1B, S1C, results for other genes were not shown). After TCGA analysis, SENP5 was observed upregulated in CRC tissues than normal tissues (Fig. 1A). SENP5 was also found significantly negatively correlated with disease-free survival rate of CRC patients (Fig. 1B). Then we investigated the possible role of SENP5 in radiotherapy resistance. To investigate the clinical relevance of SENP5, 80 patients with locally advanced rectal cancer, who received radiotherapy were divided into radiosensitive group (TRG 0–1) and radioresistant group (TRG 2 and TRG 3) based on tumor regression grade (TRG) [24]. Then the IHC staining in tissue array and quantitative analysis showed that SENP5 was significantly upregulated in the radioresistant tumors (Fig. 1C and D), which was further confirmed in tumor tissues through RT-PCR assay (Fig. 1F). The overall survival of patient with high SENP5 expression was significantly lower than those with low SENP5 expression after radiotherapy (Fig. 1E). Moreover, we checked the protein and mRNA expressions in colorectal cancer cells and normal intestine cells. Elevated expressions of both SENP5 protein and mRNA were observed in cancer cells than normal cells (Fig. 1G, Fig. S1D). CRC cells with high SENP5 expression (HT29, RKO) also showed increased resistance to ionizing radiation (IR) than those with low SENP5 expression (LOVO, SW480) (Fig. 1H). Together, our findings identified an important radioresistant gene, SENP5, which could be a potent target for overcoming radiotherapy resistance.

### Knockdown of SENP5 increased radiosensitivity both in vitro and in vivo

**Fig. 2:**
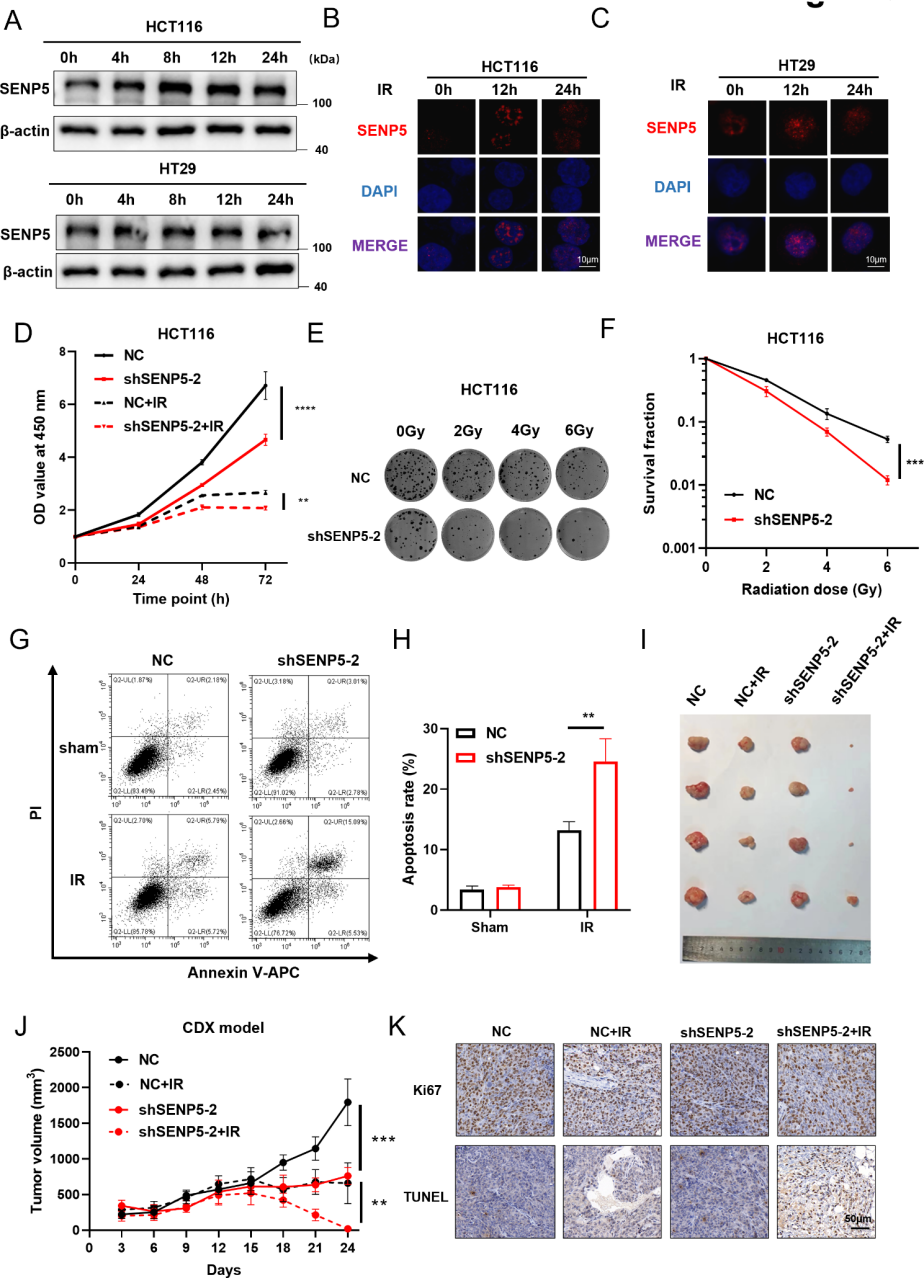
knockdown of SENP5 increased radiosensitivity in vitro and in vivo. **A** Western blotting analysis of SENP5 in irradiated HCT116 and HT29 cells at 8 Gy. **B-C** IF staining of SENP5 at different time after irradiation (8 Gy) in HCT116 and HT29 cells. Scale bar = 10 μm. **D** Cell viability analysis in irradiated HCT116 cells with NC or SENP5 knockdown transfection. ****P < 0.0001, **P < 0.01 Vs NC transfected cells. **E-F** Colony formation analysis of radiosensitivity in HCT116 cells with NC and SENP5 knockdown cells after 0, 2, 4 and 6 Gy irradiation. ***P < 0.001 Vs NC transfected cells. **G-H** Cell apoptosis assay detected with a Annexin V/PI double staining method. **P < 0.01 Vs NC transfected cells. **I** Representative images of tumors derived from NC and SENP5 knockdown cells after local irradiation. **J** Growth curve of cell derived xenograft (CDX) in tumors derived from NC and SENP5 KD cells. ***P < 0.0001 Vs relative NC groups. **K** Immunohistochemistry staining of TUNEL and Ki67 in tumor tissues isolated from NC and SENP5 knockdown tumors after irradiation

Then we determined the expression and mobilization as potential responses of SENP5 to IR. It was found that SENP5 expression was increased in a time dependent manner after irradiation in HCT116 and HT29 cells (Fig. 2A). IR also induced foci formation of SENP5 in nucleus (Fig. 2B). To investigate the influence of SENP5 on cellular radiosensitivity, we established SENP5 stably knockdown cells in both HCT116 and HT29 cells (Fig. S2A), and SENP5 knockdown cell lines constructed by shRNA-1 and shRNA-2 were selected for cellular experiments. Knockdown of SENP5 significantly inhibited cell proliferation in both irradiated and unirradiated cells (Fig. 2D, S2B, S3A, S3B). SENP5 knockdown cells showed significantly reduced colony formation capacity in irradiated HCT116 and HT29 cells (Fig. 2E F; S2C, S2D, S3C, S3D). We also found more radiation-induced cell apoptosis in SENP5 knockdown cells (Fig. 2G H; S2E, S2F, S3E, S3F). As a sequence of unrepaired DNA damage, less G2/M cell cycle arrest was also detected in SENP5 knockdown cells, compared with that in NC transfected cells (Fig. S2G-I, S3G, S3H). The above results showed that both shRNA-1 and shRNA-2 constructed SENP5 knockdown colorectal cancer cell lines could significantly inhibit the growth inhibition of tumor cells after irradiation. Among them, the knockdown effect of shRNA-2 on SENP5 protein was more obvious (Fig. 2A), so the cell line constructed by shRNA2 was used for subsequent experiments. To further explore the role of SENP5 in vivo, we monitored the sensitivity to radiotherapy by using xenografts established with SENP5 NC and SENP5 KD HCT116 cells in nude mice. Tumor growth was significantly inhibited in SENP5 knockdown cells combined with local irradiation, compared with single radiation groups (Fig. 2I). Consistent reduction in SENP5 knockdown cells derived tumors was observed in tumor size (Fig. 2J). Moreover, SENP5 knockdown led to a significant reduction in the number of proliferation cells staining for Ki67 and increased the number of apoptotic cells for TUNEL (Fig. 2K, S2J, S2K). The in vitro and in vivo data above suggesting SENP5 could be a critical target for overcoming radioresistance in CRC cells.

### SENP5 is indispensable for DNA damage repair in a HR bias manner

**Fig. 3:**
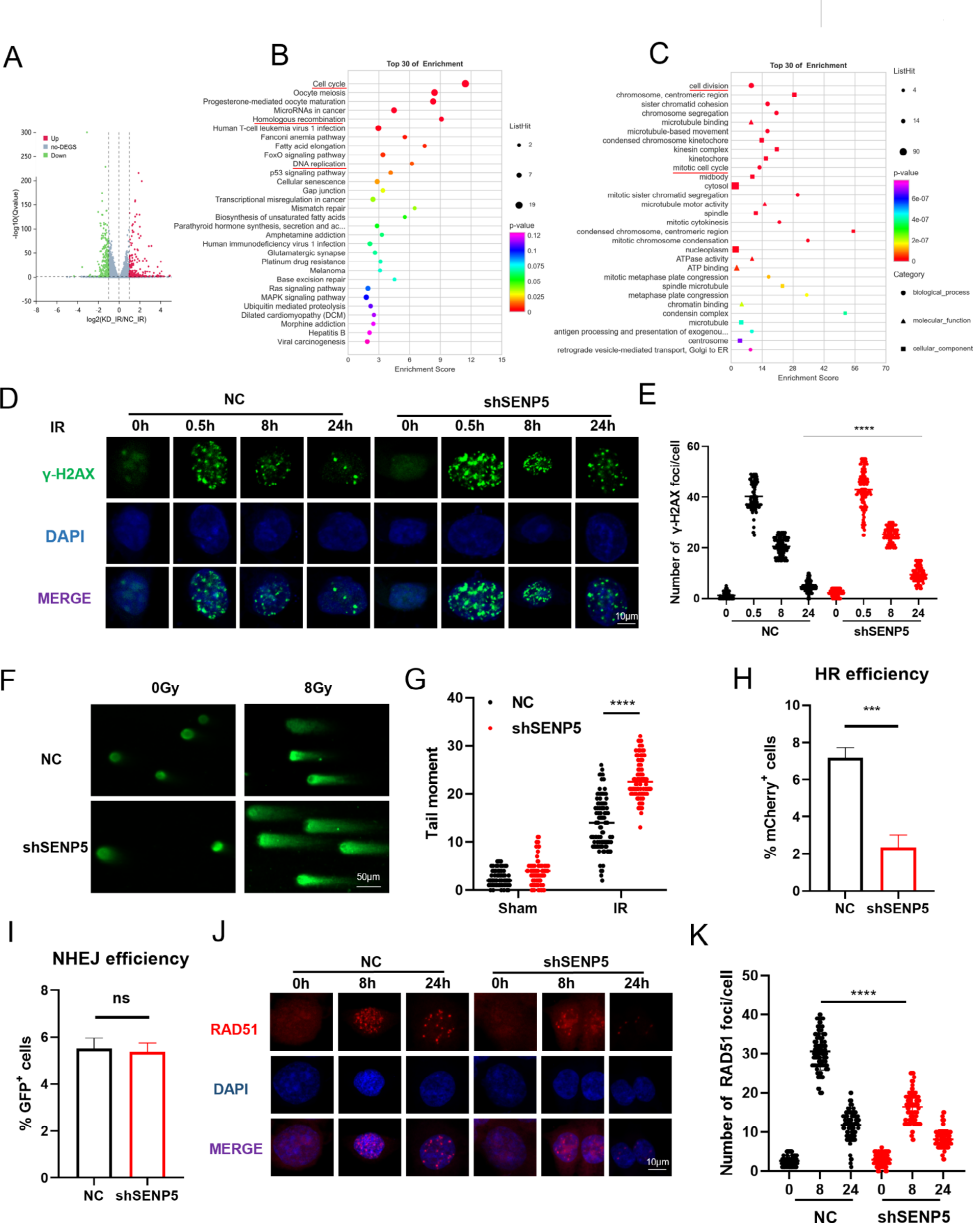
SENP5 is essential for HR-mediated DNA damage repair. **A** Volcano plot of differentially expressed genes in irradiated NC and SENP5 knockdown HCT116 cells (P < 0.05, |log2FC|≥1). **B-C** KEGG and GO enrichment of downregulated genes. **D-E** IF staining and quantitative analysis of γH2AX foci in NC and SENP5 knockdown HCT116 cells after 5 Gy irradiation. Scale bar = 10 μm. ***P < 0.001 Vs relative NC groups. **F** Representative images of comet assay in irradiated NC and SENP5 knockdown HCT116 cells. scale bar = 50 μm. **G** Tailed moment analysis of comet assay. ****P < 0.0001 Vs relative NC groups. **H-I** NHEJ and HR reporter analysis of Isel mediated DNA damage detected by flow cytometry assay. ***P < 0.001 Vs relative NC groups. **J-K** IF staining and quantitative analysis of Rad51 foci in NC and SENP5 knockdown cells after 5 Gy irradiation. Scale bar = 10 μm. ****P < 0.0001 Vs relative NC groups

To investigate the global influence of SENP5 on transcriptome, we performed RNA sequencing in NC and SENP5 KD cells after irradiation. Compared with NC group, 252 genes were upregulated and 255 genes were downregulated (Fig. 3A). Usually, radiation induces downregulation of a series of critical genes that are responsive for cellular resistance or mediating cell death. Further GO and KEGG analysis revealed that downregulated genes are mainly involved in DNA damage repair related pathways, including cell cycle, homogeneous recombination, DNA replication, Fanconi anemia pathway, mismatch repair and base excision repair (Fig. 3B C). GSEA analysis also suggested that the expression of genes related to cell cycle, DNA replication and homologous recombination repair was down-regulated in SENP5 knockdown cells (Fig. S4A-C). The core mechanism of radiotherapy is inducing abundant DNA damages, we continued to determine the influence of SENP5 on DNA damage repair. As a classic marker of DNA double strand break, the number of γH2AX foci per nucleus remained significantly higher in SENP5 KD cells than that in NC cells (Fig. 3D and E). The overall DNA damages shown by comet assay were also observed unrepaired in SENP5 KD cells after irradiation (Fig. 3F and G). To distinguish which repair pathway SENP5 mainly involved, we utilized a NHEJ and HR reporter assay as previously reported [25]. Through flow cytometry analysis, it was found that HR repair efficacy was significantly inhibited in SENP5 KD cells, instead of NHEJ pathway (Fig. 3H and I). This finding was further confirmed with the significantly reduced Rad51 foci in SENP5 KD cells, while the number of 53BP1 foci was normal (Fig. 3J K; S4D, S4E). To interrogate the HR pathway, cells were harvested at different time points after irradiation. Through a western blot analysis, it was found that knockdown of SENP5 inhibits the phosphorylation of key HR repair proteins (ATR, CHK1, CHK2) (Fig. S4F, S4G). Furthermore, we further stained Rad51 and p-CHK1 in tumor tissues of the CDX model, and observed the reduction of these two factors in tumors derived from SENP5 KD cells (Fig. S4H-J). These findings suggest that SENP5 is indispensable for efficient DNA damage repair through HR mechanism, which could provide novel mechanism for DNA damage relative cancer resistance.

### DeSUMOylation function of SENP5 was required for efficient HR repair and cell survival

**Fig. 4:**
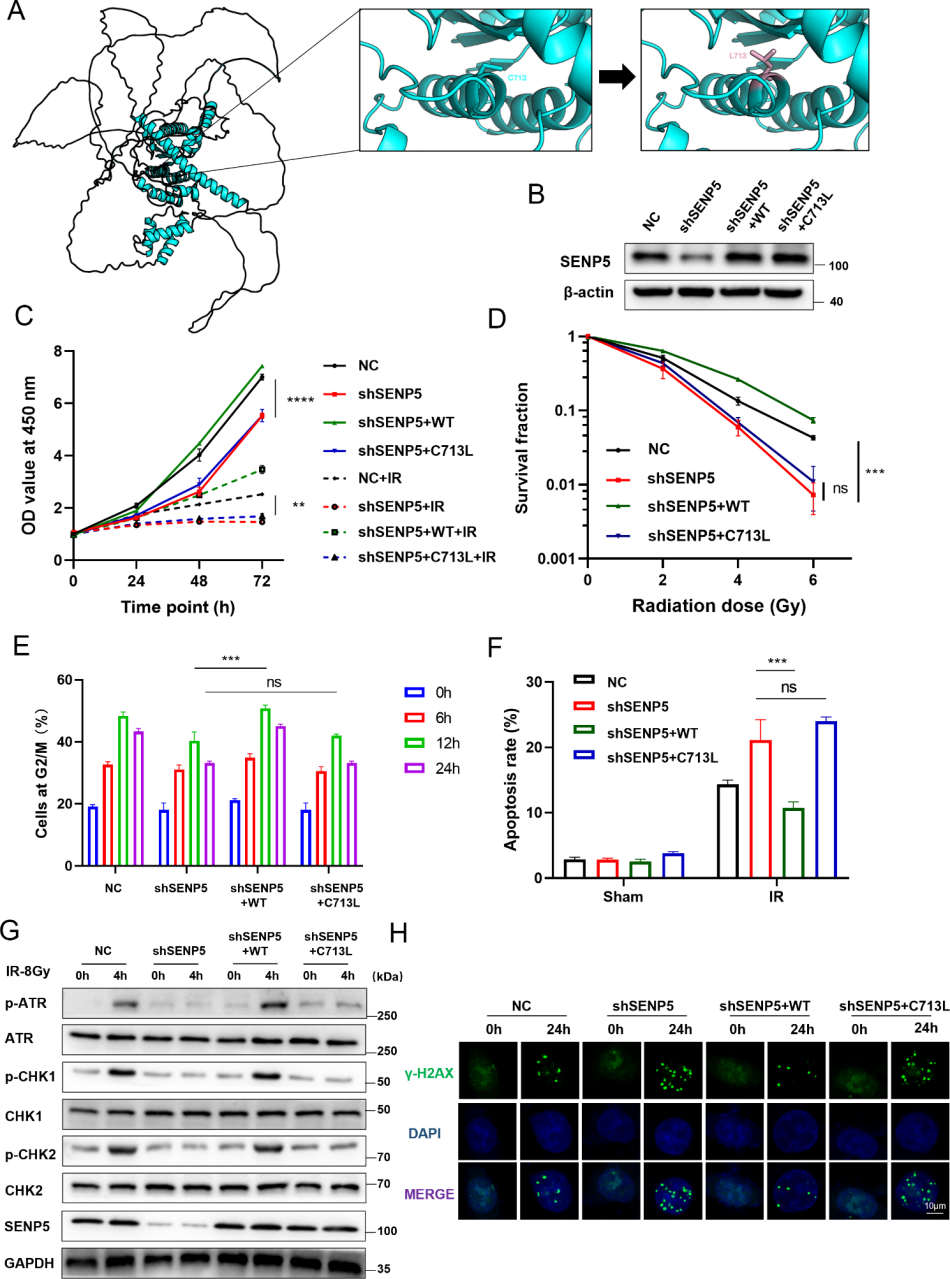
DeSUMOylation function of SENP5 is critical for its role in HR repair and radioresistance. **A** Schematic structure of SENP5 and the location of C713 site. **B** Re-expression of SENP5-wt and SENP5 C713L mutant in SENP5 knockdown cells. **C-D** Cell viability assay and colony formation analysis of with SENP5 knockdown HCT116 cells rescued with wild-type and C713L mutant SENP5. ****P < 0.0001, ***P < 0.001, **P < 0.01, Vs relative SENP5-wt rescued groups. **E** G2/M cell cycle arrest were detected with flow cytometry in SENP5 knockdown cells rescued with different mutants. Ns, non-significance, ***P < 0.001, Vs relative SENP5-wt rescued groups. **F** Cell apoptosis was detected in cells rescued with different mutants. Ns, non-significance, ***P < 0.001, Vs relative SENP5-wt rescued groups. **G** Activation of ATR related HR repair was detected by Western blotting assay in cells rescued with SENP5-wt and C713 mutant. **H** DNA damage repair efficacy were determined with γH2AX foci in SENP5 cells rescued with SENP5-wt and C713 mutant

To characterize the exact function of SENP5 accounting for HR repair and radioresistance, we constructed vectors expressing SENP5 wild type (wt) and SENP5 C713L mutant (no deSUMOylation function) in SENP5 KD HCT116 cells (Fig. 4A and B). At first, we determined cellular radiosensitivity with the following four cell lines: NC, SENP5 KD, SENP5 KD + SENP5 wt, SENP5 KD + SENP5 C713L. The CCK-8 assay showed that SENP5 wt expression effected promoted the cell proliferation of SENP5 KD cells, while no significant difference was found in SENP5KD and SENP5 C713L rescue cells (Fig. 4C). In clonogenic assay, re-expression of SENP5 wt, instead of SENP5 C713L mutant, significantly increased cellular resistance to IR (Fig. 4D). Consistent data was also found in cell cycle and apoptosis assays, no significant difference was observed between SENP5 KD and SENP5 C713L mutant groups (Fig. 4E F). As for the HR repair pathway, we found that SENP5 wt expression rescued the phosphorylation of ATR, CHK1 and CHK2 after irradiation, while no obvious change was found in SENP5 C713L mutant expressing cells (Fig. 4G). The rescue effects of unrepaired DNA termed by γH2AX foci was also found in SENP5 wt expressing cells, but not C713L mutant (Fig. 4H). These data demonstrated that the deSUMOylation function is the core mechanism of SENP5 mediated HR repair the radioresistance.

### Proteomic quantification of lysine SUMOylation in HCT116

**Fig. 5:**
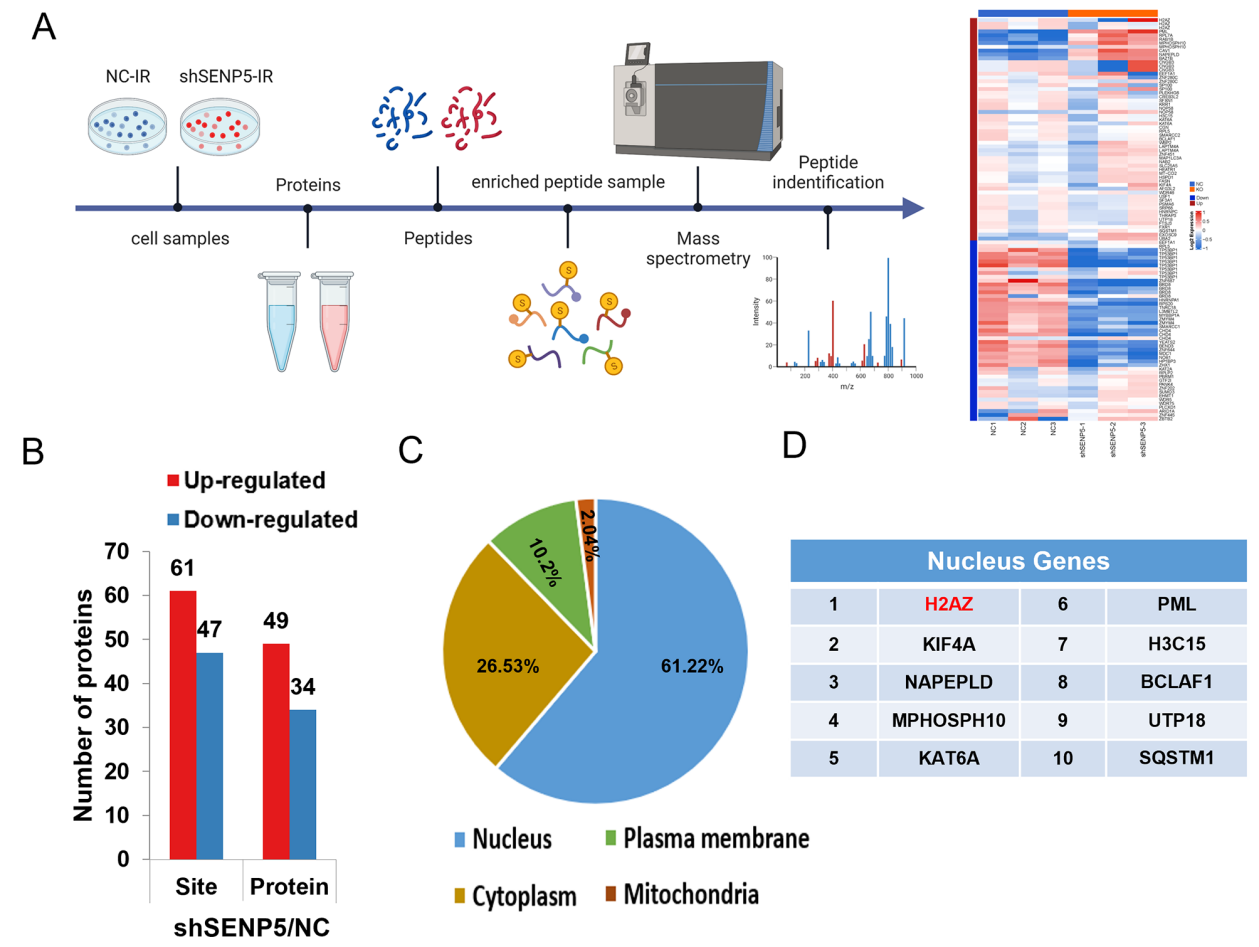
The protein SUMOylation landscape significantly differs between NC and SENP5 KD cells. **A** Flow chart of screening of deSUMOylation substrates through a SUMO-peptide pulldown assay. **B** Summary of differentially quantified sites and proteins (|log2FC| ≥1.5). **C** Subcellular localization of the identified proteins. **D** Top ten differential genes localized in the nucleus

To screening for the potential deSUMOylating targets of SENP5, we performed SUMO-protein pulldown and mass spectra assays in NC and SENP5 KD HCT116 cells with SUMO specific peptide (Fig. 5A). Applying a criteria of |log2FC| ≥1.5 and P < 0.05, the SUMOylation of 49 proteins were identified upregulated in irradiated SENP5 KD cells, while 34 proteins were downregulated (Fig. 5B). The samples were divided into four groups according to the quantitative ratio of lysine SUMOylation sites: Q1 (< 0.5), Q2 (0.5 ~ 0.667), Q3 (1.5 ~ 2), and Q5 (> 2) (Fig. S5A). The results of GO pathway enrichment cluster analysis involved molecular function, biological processes and cellular components on the four groups are shown in Fig. S5B-D. Further, analysis of the SUMOylation sites revealed that the K motif showed a strong preference for isoleucine (I) or Valine (V) in the − 1 positions, as well as for glutamic acid (E) in the + 2 position (Fig. S5E, S5F). Based on the above research results, we focused on the upregulated proteins due to the deSUMOylation function of SENP5. Further advanced analysis for 49 quantifiable SUMOylation proteins has shown that these proteins were distributed in the nucleus, cytoplasm, plasma membrane and mitochondria (Fig. 5C). Since genes localized in the nucleus are most closely related to DNA damage repair, we noticed H2AZ, the first-ranked gene in the nucleus, which may be the target of SENP5 (Fig. 5D).

### H2AZ was identified as a direct target of SENP5

**Fig. 6:**
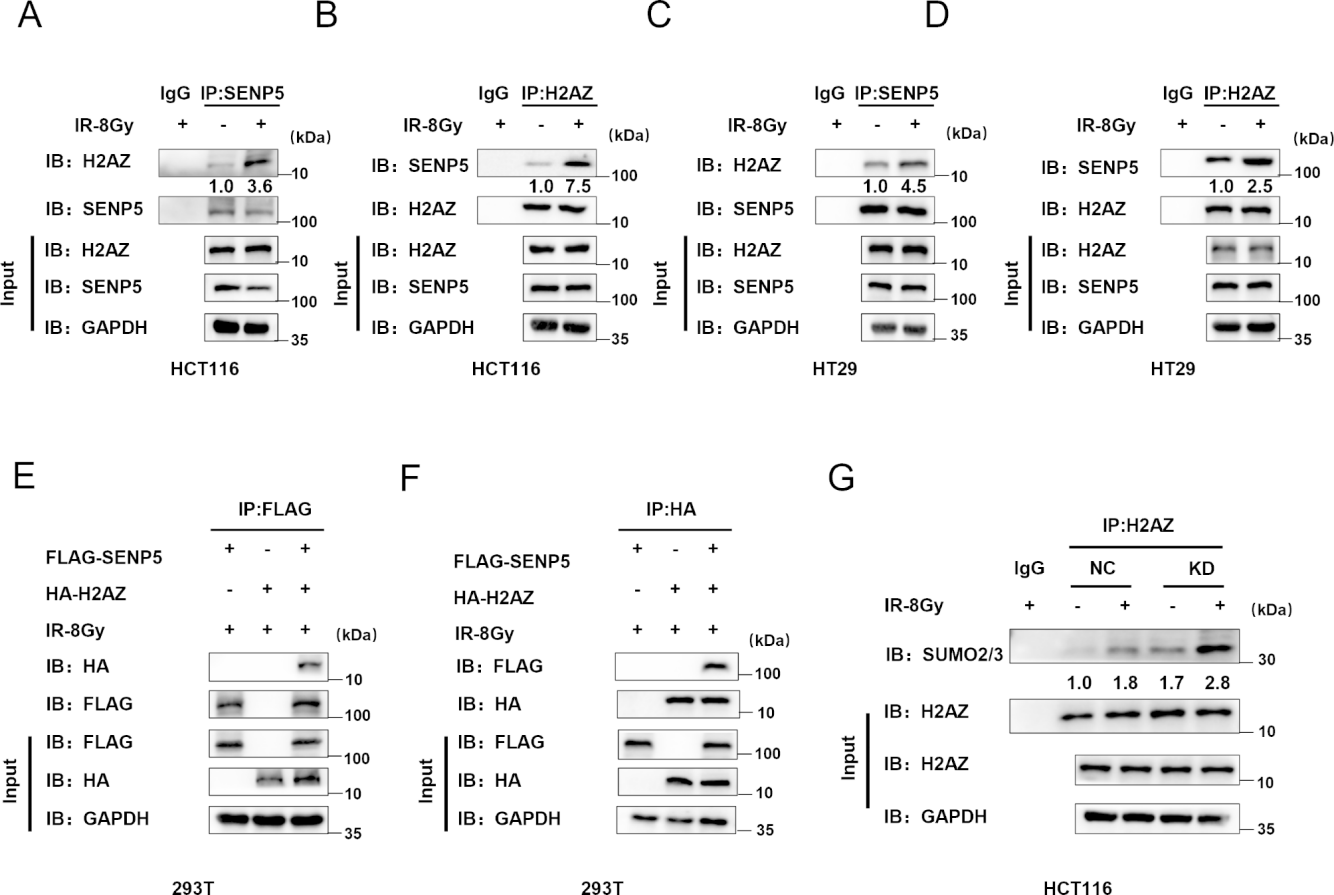
H2AZ is a direct deSUMOylation substrate. **A-D** SENP5 or H2AZ was immunoblotted in protein complex immunoprecipitated with H2AZ or SENP5 antibody in HCT116 cells (A-B) and HT29 cells (C-D), respectively. **E-F** Interaction of SENP5 and H2AZ were further validated in HEK-293T cells transfected with Flag-tagged SENP5 and HA-tagged H2AZ. **G** SUMO2/3 were detected upregulated in SENP5 knockdown cells with protein immunoprecipitated with H2AZ antibody

H2AZ, a key upstreaming factor of chromatin remodeling and DNA damage repair, remained highly SUMOylated in SENP5 knockdown cells by SUMO modification analysis. To investigate the role of H2AZ in SENP5-mediated radioresistance, we constructed H2AZ knockdown cells with H2AZ-siRNA (Fig. S6A). Subsequently, we overexpressed SENP5 in NC and siH2AZ HCT116 cells, respectively. Clone formation assay revealed that overexpression of SENP5 in cells with knockdown of H2AZ could not increase radioresistance (Fig. S6B), which revealed that H2AZ is an important target for SENP5. Then we determined the potential regulation of SENP5 on H2AZ through co-immunoprecipitation (Co-IP) assays. In HCT116 cells, H2AZ was observed upregulated after irradiation in protein lysis immunoprecipitated with SENP5 specific antibody (Fig. 6A). Inversely, SENP5 was also immunoprecipitated by H2AZ antibody (Fig. 6B). The direct interaction of SENP5 and H2AZ was also observed in irradiated HT29 cells (Fig. 6C and D). Co-IP data for exogenous expressed Flag-SENP5 and HA-H2AZ in HEK-293T cells further confirmed the direct interaction (Fig. 6E F). Finally, we detected the post-translational modifications of H2AZ affected by SENP5 through IP assay. The data showed an increase of SUMOylation of H2AZ in SENP5 KD cells (Fig. 6G). Here we identified H2AZ as a novel deSUMOylating target of SENP5 through direct interactions.

### SUMOylation of H2AZ plays critical role in SENP5 mediated HR repair

**Fig. 7:**
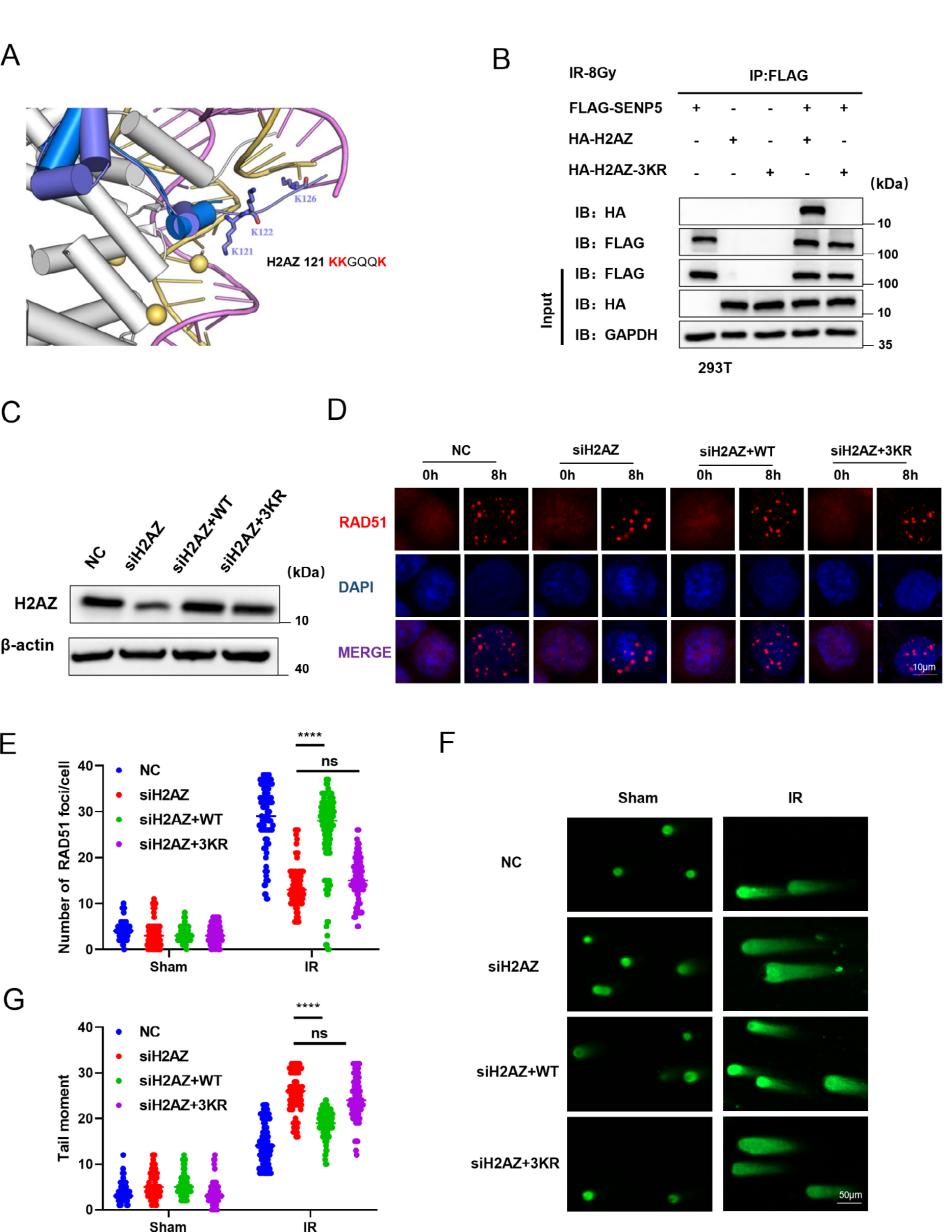
SUMOylation of H2AZ play critical role in SENP5 mediated HR repair. **A** 3D structure of H2AZ was obtained from the I-TASSER server. And K120, K121 and K126 sites were observed to be responsible for DNA binding. **B** H2AZ-wt and H2AZ-K3R was constructed and expressed into H2AZ knockdown cells. **C** Activation of ATR related HR repair was detected by Western blotting assay in cells rescued with H2AZ-wt and K3R mutant. **D-E** Rad51 foci as key marker of HR repair was detected with IF assay in cells rescued with different mutants. Scale bar = 10 μm. ****P < 0.0001 Vs the H2AZ-wt group. **F-G** DNA damage were evaluated with comet assay in cells rescued with different mutants. ****P < 0.0001 Vs the H2AZ-wt group

The critical role of SUMOylation balance of H2AZ promotes us to investigate its role in HR repair-mediated cancer resistance. Based on SUMO-proteomic mass spectrometry, the SUMO modification at lysine (lysine 121, 122, and 126) of H2AZ increased in SENP5 knockdown cells (Fig. S7A-C). We simulated the structure of H2AZ and surprisingly identified that the three SUMOylation sites (K121, K122, K126) revealed by mass assay are within the DNA binding domains in C-terminal (Fig. 7A). Furthermore, the SUMOylation sites of H2AZ was aligned with those from different species (Fig. S5D). Therefore, we mutated all lysine residue to an arginine (3KR: K121R, K122R, and K126R). Co-IP data for exogenous expressed Flag-SENP5 and HA-H2AZ or HA-H2AZ 3KR in HEK-293T further confirmed mutating H2AZ SUMOylation sites in H2AZ impaired its interaction with SENP5 (Fig. 7B). Then we established cell lines in H2AZ knockdown HCT116 cells expressing the mutant of these 3 lysine (K) to arginine (R) (3KR), accompanying wt H2AZ (Fig. 7C). The average number of Rad51 foci was also less than that in the wt-H2AZ expressing cells, suggesting defects in HR repair (Fig. 7D and E). The cellular radiosensitivity was also increased in cells expressing H2AZ 3KR mutant (Fig. 7F and G). These findings revealed that the SUMOylation of H2AZ was critical for the SENP5-mediated HR repair of DNA damage.

### Targeting SENP5 increased the therapeutic efficacy of radiotherapy in both patient-derived organoids (PDO) and xenograft (PDX) models

**Fig. 8:**
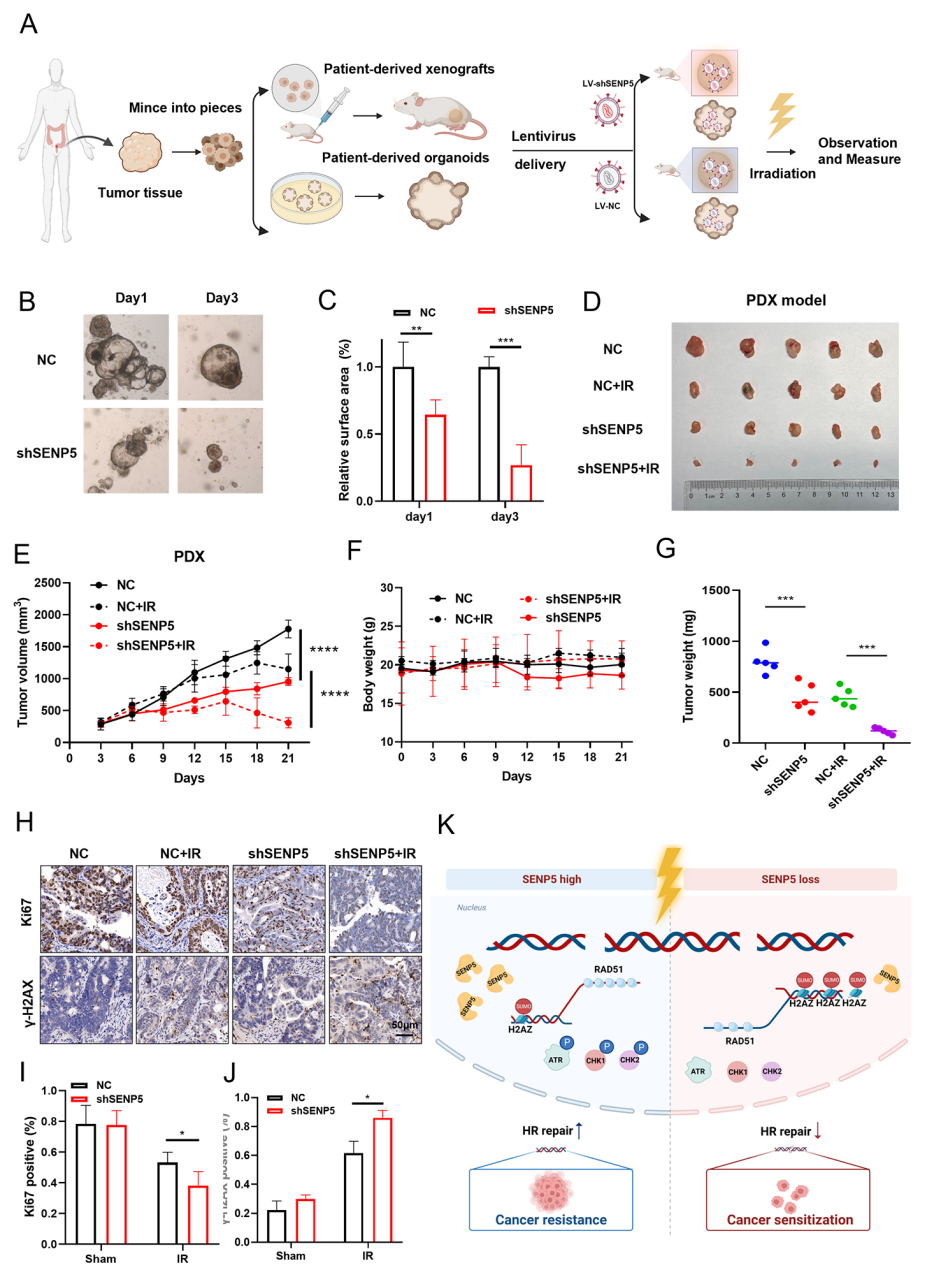
Targeting SENP5 improved the efficacy of radiotherapy in both PDO and PDX preclinical models. **A** Flow chart of the establishment of PDO and PDX from clinical patients. After then the PDO and PDX were exposed to indicated doses of radiation and subjected to next experiments. **B** Representative images of PDO transfected with lenti-virus packaged NC or SENP5 shRNA after exposed to 8 Gy irradiation. **C** Quantitative analysis of organoids in different groups. **P < 0.01, *P < 0.05 Vs the NC group. **D** Representative images of tumors isolated from PDX bearing mice in NC and SENP5 knockdown groups at the end point of observation after local irradiation. **E** Growth curves were generated with tumor sizes up to day 21 post-irradiation. ***P < 0.001, *P < 0.05 Vs the NC group. **F** Body weight of PDX tumor bearing mice were monitored every three days. **G** Tumor weights were measured in different groups. **H-J** IHC staining and quantitative analysis of γH2AX and Ki67 positive area in tumor tissues isolated in different groups. *P < 0.05 Vs the NC group. **K** Hypothetical model: SENP5 promotes deSUMOylation of H2AZ to promotes its removal and the recruitment of HR repair factors, which confers cancer radioresistance

We finally applied two preclinical models including PDO and PDX model to investigate the possibility of targeting SENP5 in CRC treatment. First of all, PDO and PDX models were established by using the tumor tissues surgically resected from rectal cancer patients in Changhai hospital (Fig. 8A). In PDO experiments, the organoids were transfected with lenti-virus expressing NC and shSENP5 and exposed to 8 Gy ionizing radiation. After monitoring the organoids growth, it was found that SENP5 knockdown combined with irradiation significantly inhibited the growth of PDO (Fig. 8B C). In PDX experiments, lenti-virus expressing NC and shSENP5 were intratumorally injected into tumor. When tumor size reached 500mm^3^, tumor bearing mice were exposed to local irradiation, and tumor growth as well as body weight were measured every three days. Our data showed that SENP5 knockdown combined with local irradiation significantly inhibited tumor growth (tumor volume) (Fig. 8D and E). The body weight of mice in each group were unaffected (Fig. 8F). Tumor weight in the SENP5 KD groups was also reduced, compared with the single radiation group (Fig. 8G). Immunohistochemical analysis revealed that the expression of γ-H2AX was significantly elevated and the percentage of Ki67-positive cells was significantly decreased in SENP5 knockdown tumor cells after irradiation (Fig. 8H-J). Taken together, these findings provide important evidence that targeting SENP5 could be an effective strategy for CRC treatment. The overall working model of this study was summarized as a flowchart in Fig. 8K.

## Discussion

The cellular response to DNA damage is an important determinant of cancer progression and cancer outcome in radiation therapy [9]. Identifying novel molecular events such as PTM, including phosphorylation, ubiquitination, SUMOylation etc., that regulate DNA damage signaling and DNA repair will help to better understand the mechanisms of cancer resistance to of radiotherapy [26, 27]. In our present study, we firstly screened and characterized the role of SENP5 in radiotherapy resistance in aspects of cellular models, cell derived xenograft models, PDO and PDX preclinical models. Mechanistically, SENP5 was demonstrated to promote HR mediated DNA repair through deSUMOylation of H2AZ at three lysine sites, which affects the recruitment of the downstream HR factors. High expression of SENP5 was also found in patient resistant to radiotherapy through tissues array obtained from clinical patients.

SUMOylation is mainly found in the nucleus and play a key role in many nuclear processes such as gene expression, genome stability, DNA damage response, protein transport and cell cycle control [17]. In the kinetics of SUMOylation balance, the SENP family mainly accounts for removing SUMO proteins to regulate protein function, which were also reported to be prognostic value with critical functions [23, 28]. For instance, SENP1 and SENP2 genes polymorphisms are associated with different hormone receptor status, lymph node status and tumor grades in breast cancer [29]. Knockdown of SENP1 delays mitotic progression due to a failure of mid-term sister chromatid separation [30]. Overexpression of SENP2 resulted in prometaphase arrest due to a defect in the microtubule engine protein CENP-E targeting the kinetochore [31]. Recently, SENP3 was demonstrated to regulate DNA-PKcs mediated DNA damage repair through deSUMOylation of TIP60 [32]. SENP5 was found upregulated in several types of cancer, including oral squamous cell carcinoma [33], hepatocellular carcinoma (27,649,656), and polymorphisms observed in non-small cell lung cancer [34]. Our findings demonstrated that SENP5 was upregulated in CRC, and correlated with poor outcomes. More importantly, our data from tissue array revealed the significant prognostic value patient resistant to radiotherapy, which could be applied to distinguish those tolerant to priority to surgery.

The main mechanism of radiotherapy together with large number of chemotherapy is inducing unfavorable DNA damages. Our RNA sequencing data also revealed that SENP5 affected several signaling pathway related to DNA damage repair. SENP5 was also found to deSUMOylate AURKA to regulated mitosis [35]. It was also reported that SENP5-depleted HepG2 cells exhibited hypersensitivity to DNA damage treatment in HCC [36]. In our CRC cells, we firstly revealed that SENP5 expression level is correlated with radioresistance, and further characterized the role of SENP5 in HR-mediated DNA damage repair. The defect of HR repair was supported with multiple step evidence, including NHEJ/HR reporter assay, Rad51 foci, as well as IHC staining in CDX tissues. HR repair occurs mainly in the S phase and is also the main way for cells to repair damaged DNA precisely. Many basic studies have reported that inhibition of HR repair significantly inhibits the growth of tumor cells. Therefore, many clinical trials have investigated HR repair as a potential target for tumor therapy, including ATR, ATM, and Rad51 [37, 38]. The critical role of SENP5 in HR repair provides great opportunity for application in cancer therapy.

As a SUMO-specific protease, SENP5 is mainly localized in the nucleus, and mainly participates in the regulation of protein SUMOylation during the following two processes: to participate in the initiation processing of SUMO precursors, generating a bisglycine motif at its C-terminus for ligation reactions; to remove SUMO molecules from high molecular weight SUMO conjugates by isopeptidase activity (deSUMOylation), which is the most important function of SENP5 [39]. We further demonstrated that loss of function mutant of SENP5 deSUMOylation failed to rescue DNA damage repair and cell survival. To uncover the critical substrates during HR-mediated DNA damage repair was critical for uncover its underlying mechanism. Till now, several substrates of SENP5 have been identified, including DRP1, RanGAP1 ATRIP [36, 40, 41]. However, these molecules could not explain the defects in efficient HR repair.

In our data of SUMO-specific protein mass spectra, H2AZ was identified highly SUMOylated in SENP5 knockdown cells. Further data revealed that H2AZ is a direct deSUMOylation substrate of SENP5. As a variant of H2A family, H2AZ mainly located in euchromatin and heterochromatin [42]. Several studies have demonstrated that H2AZ is required for the early activation of activation of DNA damage repair. H2AZ has been observed to translocate to DSB sites and regulate transcription, chromatin remodeling as well as the recruitment of other DNA repair factors [43, 44]. After DSB repair, H2AZ was removed by the nucleosome remodeling enzyme INO80 and initiated the downstream signaling of DNA repair [45]. Importantly, recruitment and modification of H2AZ was crucial for the activation of key HR factors including BRCA1 and Rad51 [46, 47]. Among all PTM types, ubiquitination and SUMOylation was reported as critical steps of H2AZ function [48]. Notably, obviously increased SUMOylation level was observed and related to break recruitment [49]. However, deSUMOylation of H2AZ and the specific sites have not been reported. Our data revealed a direct role of SENP5 in H2AZ deSUMOylation at three lysine sites, which regulates its chromatin binding kinetics and HR repair. Our findings provide novel insight of SENP5 mediated deSUMOylation of H2AZ into HR repair. However, there are still some questions about the SUMOylation of H2AZ that need to be further investigated, such as what is the E3 ligase that mediates SUMOylation of H2AZ, and whether there is a crosstalk between (de) SUMOylation, ubiquitination, and acetylation of H2AZ.

Our findings of SENP5 in conferring radiation resistance promotes us to investigate its clinical application potentials. Currently, patient derived organoids and xenograft models could well present the clinical features [50]. Our data showed that inhibiting SENP5 effectively suppressed organoids and tumor grow when combined with radiation. Due to the absence of immunocytes in organoids, the combined therapeutic effects could be due to impaired DNA damage repair. Moreover, more precise strategies specifically inhibiting the deSUMOylation domain of SENP5 should be developed.

## Conclusions

Our study reveals that SENP5 is a potent gene associated with radiotherapy resistance and is involved in cellular HR repair by regulating SUMO modification of H2AZ. Our findings provide a novel mechanism for radiotherapy resistance and suggest that SENP5 could be a potential target to improve radiotherapy efficacy.

### Electronic supplementary material

Below is the link to the electronic supplementary material.

Supplementary Material 1

Supplementary Material 2

Supplementary Material 3

Supplementary Material 4

## Data Availability

All relevant data not presented in the main figures or in the supplementary data are available from the authors.

## Abbreviations

ATCC: American Type Culture Collection
Co-ip: Co-immunoprecipitation
CRC: Colorectal cancer
DSBs: Double strand breaks
HR: Homologous recombination
IR: Ionizing radiation
NHEJ: Nonhomologous end joining
PBS: Phosphate-buffered saline
PDO: Patient-derived organoids
PDX: Patient-derived xenograft
PIKK: PI3K-related kinase
PTMs: Post-translational modifications
RNA-seq: RNA sequencing
SENP5: SUMO specific peptidase 5
SENPs: Sentrin-specific proteases

## References

[1] Dekker E (2019). Colorectal cancer. Lancet.

[2] Fokas E (2020). Outcome measures in multimodal rectal cancer trials. Lancet Oncol.

[3] Sedlak JC, Yilmaz OH, Roper J. Metabolism and colorectal Cancer. Annu Rev Pathol; 2022. 10.1146/annurev-pathmechdis-031521-041113 PMC9877174 36323004

[4] Chatila WK (2022). Genomic and transcriptomic determinants of response to neoadjuvant therapy in rectal cancer. Nat Med.

[5] Negri F, Aschele C (2022). Unconsolidated results of consolidation chemotherapy following short-course Radiotherapy in locally advanced rectal Cancer. J Clin Oncol.

[6] Wang XC (2019). Genome-wide RNAi screening identifies RFC4 as a factor that mediates Radioresistance in Colorectal Cancer by facilitating Nonhomologous End joining repair. Clin Cancer Res.

[7] Zhou Y, et al. A novel long noncoding RNA SP100-AS1 induces radioresistance of colorectal cancer via sponging miR-622 and stabilizing ATG3. Cell Death Differ; 2022. 10.1038/s41418-022-01049-1 PMC9883267 35978049

[8] Groelly FJ et al. *Targeting DNA damage response pathways in cancer*. Nat Rev Cancer, 2022. 10.1038/s41568-022-00535-5 36471053

[9] Huang R, Zhou PK (2021). DNA damage repair: historical perspectives, mechanistic pathways and clinical translation for targeted cancer therapy. Signal Transduct Target Ther.

[10] Jin Z, Sinicrope FA (2022). Mismatch repair-deficient Colorectal Cancer: building on checkpoint blockade. J Clin Oncol.

[11] Mekonnen N, Yang H, Shin YK (2022). Homologous recombination Deficiency in Ovarian, breast, colorectal, Pancreatic, non-small cell lung and prostate cancers, and the Mechanisms of Resistance to PARP inhibitors. Front Oncol.

[12] Ismail IH (2015). The RNF138 E3 ligase displaces Ku to promote DNA end resection and regulate DNA repair pathway choice. Nat Cell Biol.

[13] Durinikova E (2022). Targeting the DNA damage response pathways and replication stress in Colorectal Cancer. Clin Cancer Res.

[14] Iliakis GE (2021). New Players in the regulation of DNA-PK activity: Survivin joins the crowd. Cancer Res.

[15] Sharma A (2018). USP14 regulates DNA damage repair by targeting RNF168-dependent ubiquitination. Autophagy.

[16] Millan-Zambrano G (2022). Histone post-translational modifications - cause and consequence of genome function. Nat Rev Genet.

[17] Sarangi P, Zhao X (2015). SUMO-mediated regulation of DNA damage repair and responses. Trends Biochem Sci.

[18] Chang HM, Yeh ETH (2020). SUMO: from bench to Bedside. Physiol Rev.

[19] Garvin AJ (2019). The deSUMOylase SENP2 coordinates homologous recombination and nonhomologous end joining by independent mechanisms. Genes Dev.

[20] Vyas R (2013). RNF4 is required for DNA double-strand break repair in vivo. Cell Death Differ.

[21] Wu CS (2014). SUMOylation of ATRIP potentiates DNA damage signaling by boosting multiple protein interactions in the ATR pathway. Genes Dev.

[22] Gao Y (2022). SENP1 promotes triple-negative breast cancer invasion and metastasis via enhancing CSN5 transcription mediated by GATA1 deSUMOylation. Int J Biol Sci.

[23] Tokarz P, Wozniak K. *SENP proteases as potential targets for Cancer Therapy*. Cancers (Basel), 2021. 13(9). 10.3390/cancers13092059 PMC8123143 33923236

[24] Yu Y (2022). PRDM15 interacts with DNA-PK-Ku complex to promote radioresistance in rectal cancer by facilitating DNA damage repair. Cell Death Dis.

[25] Arnoult N (2017). Regulation of DNA repair pathway choice in S and G2 phases by the NHEJ inhibitor CYREN. Nature.

[26] Chatterjee S, Senapati P, Kundu TK (2012). Post-translational modifications of lysine in DNA-damage repair. Essays Biochem.

[27] Han ZJ (2018). The post-translational modification, SUMOylation, and cancer (review). Int J Oncol.

[28] Mendes AV (2016). Evaluation of the activity and substrate specificity of the human SENP family of SUMO proteases. Biochim Biophys Acta.

[29] Mirecka A, Morawiec Z, Wozniak K (2016). Genetic polymorphism of SUMO-Specific cysteine proteases - SENP1 and SENP2 in breast Cancer. Pathol Oncol Res.

[30] Maruyama T, Abe Y, Niikura T (2018). SENP1 and SENP2 regulate SUMOylation of amyloid precursor protein. Heliyon.

[31] Zhang XD (2008). SUMO-2/3 modification and binding regulate the association of CENP-E with kinetochores and progression through mitosis. Mol Cell.

[32] Han Y et al. *SENP3-mediated TIP60 deSUMOylation is required for DNA-PKcs activity and DNA damage repair* MedComm (2020), 2022. 3(2): p. e123. 10.1002/mco2.123 PMC8941250 35356800

[33] Meng Y, Li X (2021). Expression and prognosis analysis of SUMOylation regulators in oral squamous cell Carcinoma based on high-throughput sequencing. Front Genet.

[34] Nastase A (2020). Platinum drug sensitivity polymorphisms in stage III non-small cell Lung Cancer with Invasion of Mediastinal Lymph Nodes. Cancer Genomics Proteomics.

[35] Yu B et al. *SUMO proteases SENP3 and SENP5 spatiotemporally regulate the kinase activity of Aurora A*. J Cell Sci, 2021. 134(13). 10.1242/jcs.249771 34313310

[36] Jin ZL (2016). The SUMO-specific protease SENP5 controls DNA damage response and promotes tumorigenesis in hepatocellular carcinoma. Eur Rev Med Pharmacol Sci.

[37] Bradbury A (2020). Targeting ATR as Cancer Therapy: a new era for synthetic lethality and synergistic combinations?. Pharmacol Ther.

[38] Garcia MEG, Kirsch DG, Reitman ZJ (2022). Targeting the ATM kinase to enhance the efficacy of Radiotherapy and Outcomes for Cancer Patients. Semin Radiat Oncol.

[39] Hotz PW, Muller S, Mendler L (2021). SUMO-specific Isopeptidases tuning Cardiac SUMOylation in Health and Disease. Front Mol Biosci.

[40] Zunino R (2007). The SUMO protease SENP5 is required to maintain mitochondrial morphology and function. J Cell Sci.

[41] Di Bacco A (2006). The SUMO-specific protease SENP5 is required for cell division. Mol Cell Biol.

[42] Kelliher JL (2020). Histone H2A variants alpha1-extension helix directs RNF168-mediated ubiquitination. Nat Commun.

[43] Dunican DS (2002). Gene expression differences between the microsatellite instability (MIN) and chromosomal instability (CIN) phenotypes in colorectal cancer revealed by high-density cDNA array hybridization. Oncogene.

[44] Hayakawa K (2017). H2A O-GlcNAcylation at serine 40 functions genomic protection in association with acetylated H2AZ or gammaH2AX. Epigenetics Chromatin.

[45] Alatwi HE, Downs JA (2015). Removal of H2A.Z by INO80 promotes homologous recombination. EMBO Rep.

[46] Leung JW (2017). ZMYM3 regulates BRCA1 localization at damaged chromatin to promote DNA repair. Genes Dev.

[47] Waters R, van Eijk P, Reed S (2015). Histone modification and chromatin remodeling during NER. DNA Repair (Amst).

[48] Li Z (2014). Dynamics of polycomb proteins-mediated histone modifications during UV irradiation-induced DNA damage. Insect Biochem Mol Biol.

[49] Kalocsay M, Hiller NJ, Jentsch S (2009). Chromosome-wide Rad51 spreading and SUMO-H2A.Z-dependent chromosome fixation in response to a persistent DNA double-strand break. Mol Cell.

[50] Huang L et al. *PDX-derived organoids model in vivo drug response and secrete biomarkers*. JCI Insight, 2020. 5(21). 10.1172/jci.insight.135544 PMC7710298 32990680

