# Supplementary material for: SENP5 promotes homologous recombination-mediated DNA damage repair in colorectal cancer cells through H2AZ deSUMOylation
Source: J Exp Clin Cancer Res. 2023 Sep 8;42:234. doi: 10.1186/s13046-023-02789-9 (PMC10486113; doi:10.1186/s13046-023-02789-9)
Supplement: Supplementary file 2 — Supplementary Material 2 [file 13046_2023_2789_MOESM2_ESM.pdf]

# Supplementary Information

## SENP5 promotes homologous recombination-mediated DNA damage repair in colorectal cancer cells through H2AZ deSUMOylation

Tingting Liu[#1], Hang Wang[#1], Yuanyuan Chen[#1,] Zhijie Wan[#1], Zhipeng Du[2], Hui Shen[1], Yue Yu[3], Shengzhi Ma[3], Ying Xu[1], Zhuqing Li[1], Nanxi Yu[2], Fangxiao Zhang[2], Kun Cao[1], Jianming Cai[2], Wei Zhang*[1], Fu Gao*[1], Yanyong Yang*[1]

## Supplementary figures

**Figure S1**

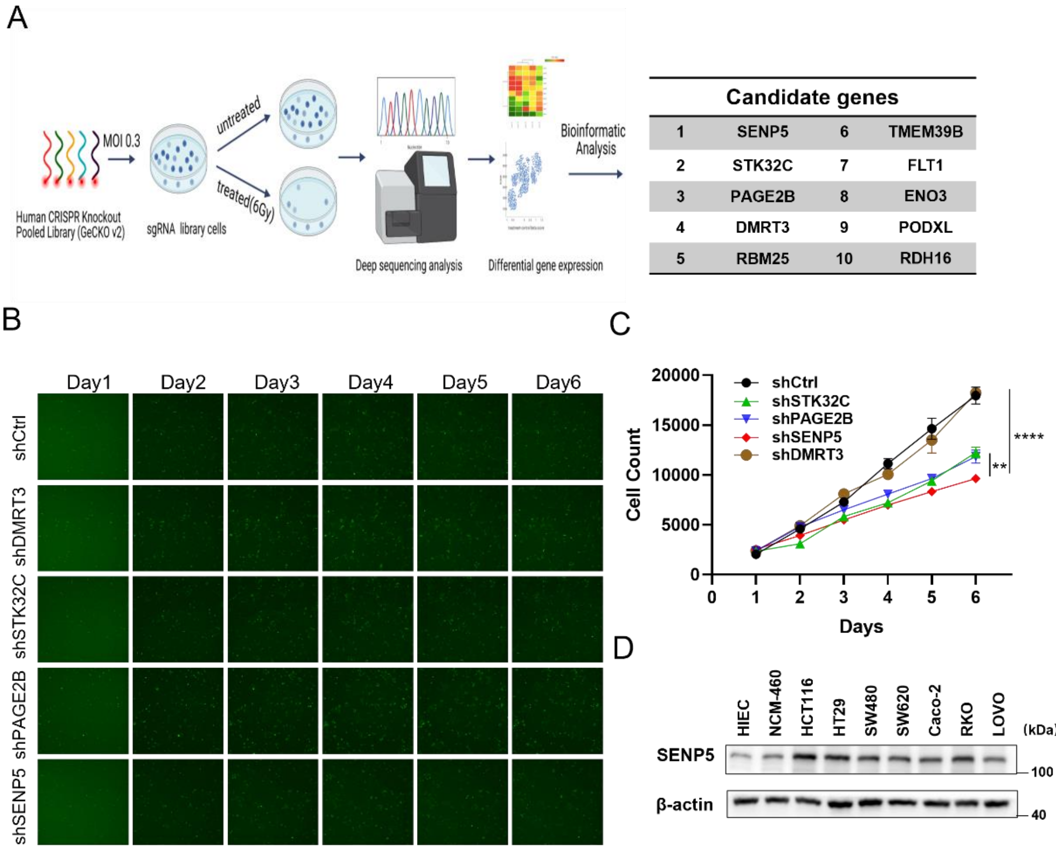


**Figure S1 Screening of radioresistant genes.**

**A** CRISPR Cas9 genomic library screening combined with bioinformatics analysis to screen radioresistant genes. **B-C** Cell proliferation was analyzed by high content screening. **D** The protein expressions of SENP5 in normal intestine and CRC cell lines.

# Figure S2

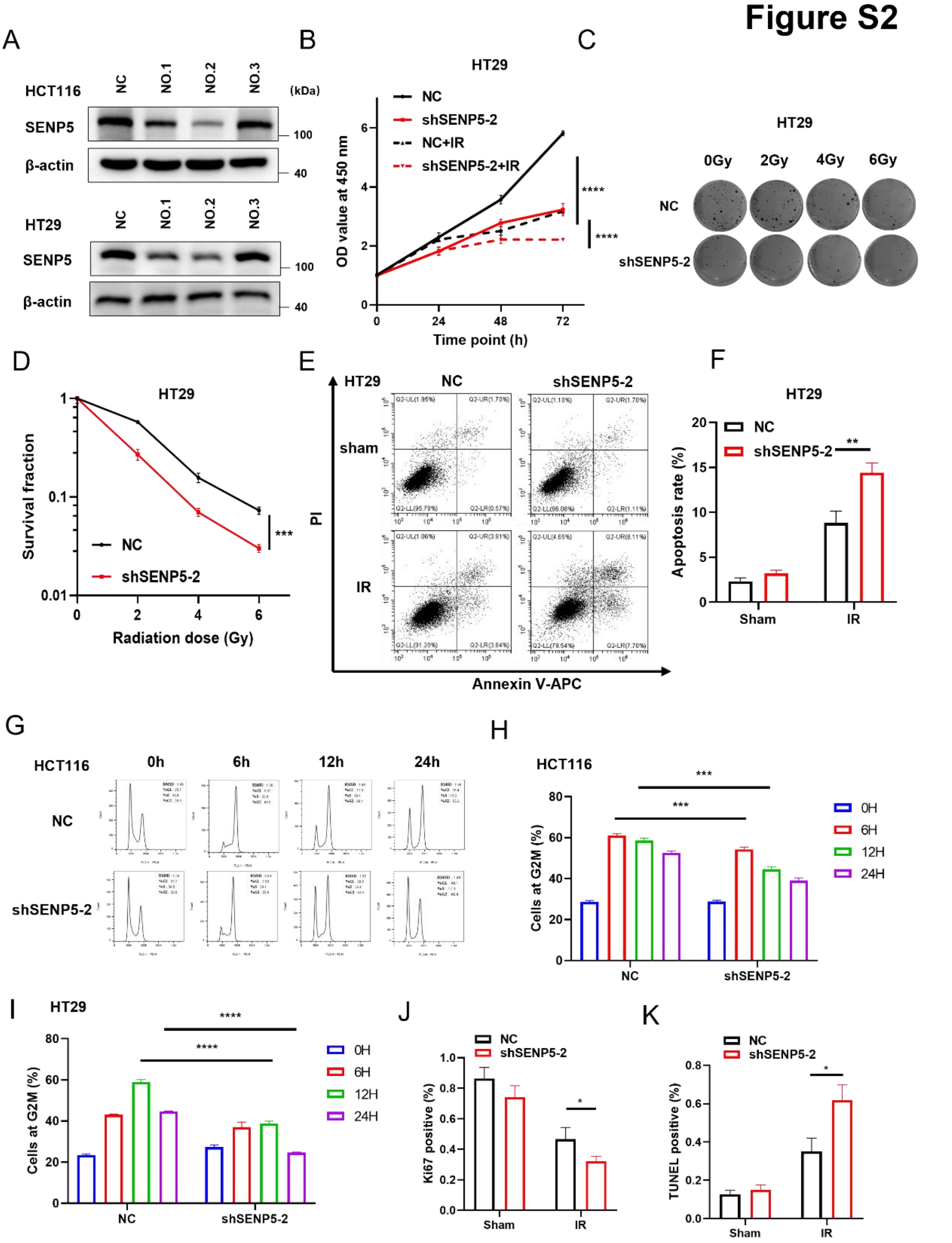


**Figure S2 Knockdown of SENP5 increased radiosensitivity in vitro and in vivo.**

**A** Knockdown of SENP5 with three independent lentivirus packaged shRNA. **B** Cell viability analysis in irradiated HT29 cells with NC or SENP5 knockdown transfection. ****P<0.0001 Vs NC transfected cells. **C-D** Colony formation analysis of radiosensitivity in HT29 cells with NC and SENP5 knockdown after 0, 2, 4 and 6Gy irradiation. ***P<0.001 Vs NC transfected cells. **E-F** Cell apoptosis assay detected with

a Annexin V/PI double staining method in HT29 cells. **P<0.01 Vs NC transfected cells. **G-I** Flow cytometry cell cycle analysis in HCT116 and HT29 cells with NC and SENP5 knockdown. ****P<0.0001, ***P<0.001 represent cells in G2M cell cycle Vs that in NC transfected cells. **J-K** Quantification of the TUNEL-positive and Ki67-positive cells. *P<0.05 Vs NC transfected cells.

# Figure S3

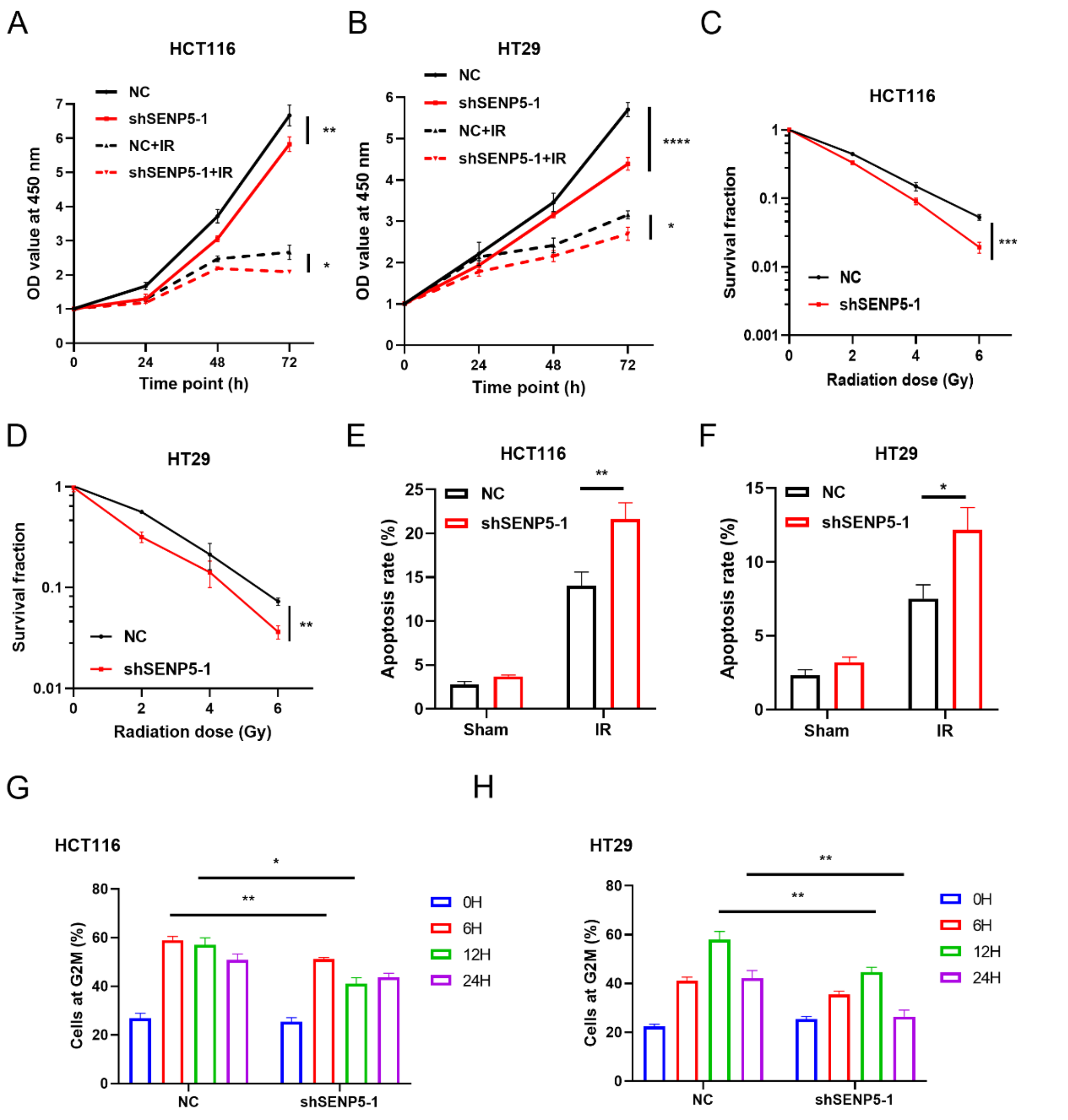


**Figure S3 knockdown of SENP5 increased radiosensitivity in vitro and in vivo.** **A-B** Cell viability analysis in irradiated HCT116 and HT29 cells with NC or SENP5 knockdown transfection. *P<0.05, **P<0.01, ****P<0.0001 Vs NC transfected cells. **C-D** Colony formation analysis of radiosensitivity in HCT116 and HT29 cells with NC and SENP5 knockdown after 0, 2, 4 and 6Gy irradiation. **P<0.01, ***P<0.001 Vs NC

transfected cells. **E-F** Cell apoptosis assay detected with a Annexin V/PI double staining method in HCT116 and HT29 cells. **P<0.01 Vs NC transfected cells. *P<0.05, **P<0.01 Vs NC transfected cells. **G-H** Flow cytometry cell cycle analysis in HCT116 and HT29 cells with NC and SENP5 knockdown. *P<0.05, **P<0.01 represent cells in G2M cell cycle Vs that in NC transfected cells.

# Figure S4

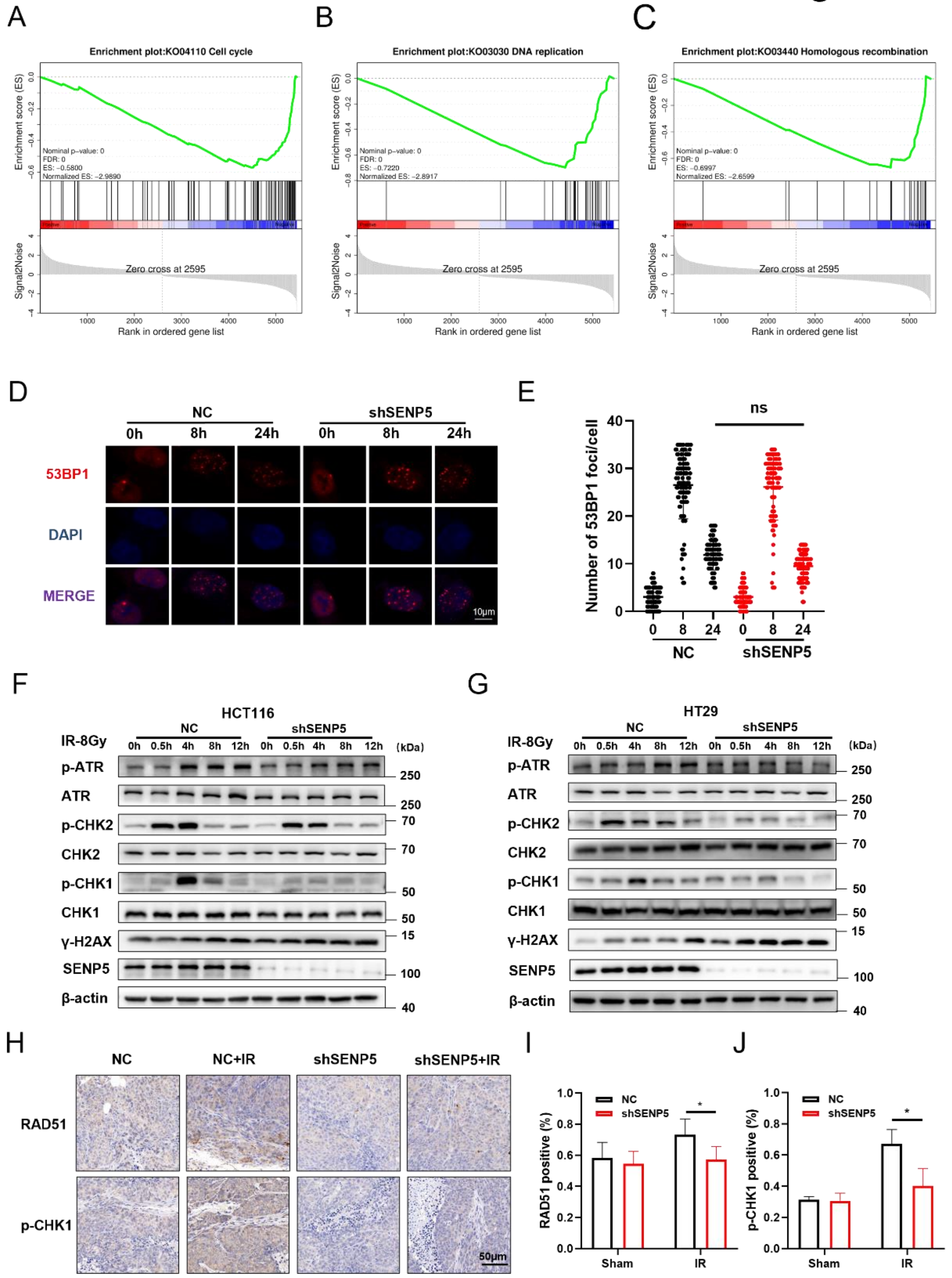


**Figure S4 SENP5 is essential for HR-mediated DNA damage repair.**

**A-C** GSEA analysis was performed based on RNA-seq results after knockdown of SENP5. **D-E** IF staining and quantitative analysis of 53BP1 foci in NC and SENP5 knockdown cells after 5Gy irradiation. Scale bar=10μm. ns (no significance) Vs relative NC groups. **F-G** Western blotting analysis of NC and SENP5 knockdown in irradiated

HCT116 and HT29 cells at 8Gy. **H-J** IHC staining of Rad51 and P-CHK1 in tumor tissues from irradiated CDX model. *P<0.05 Vs relative NC groups.

## Figure S5

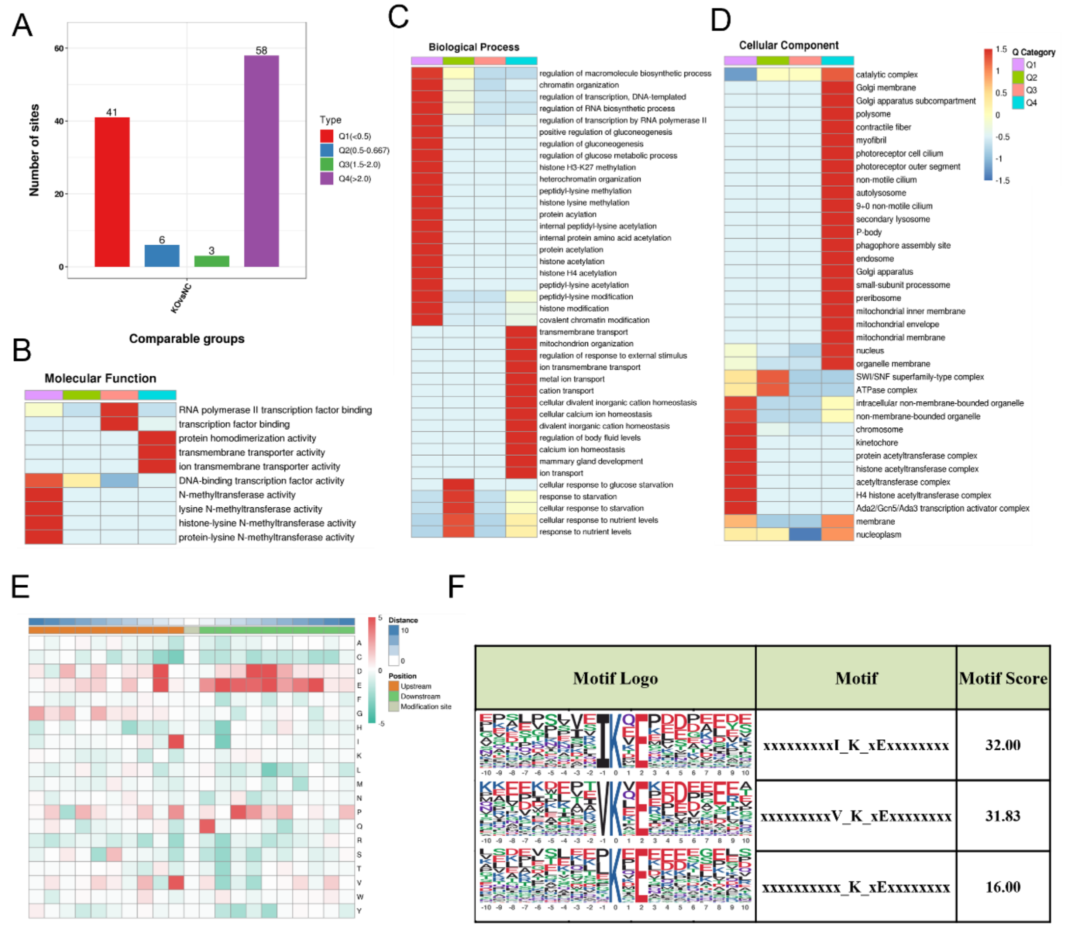


**Figure S5 The protein SUMOylation landscape significantly differs between NC and SENP5 KD cells.**

**A-D** GO pathway enrichment analysis of four groups of samples, Q1 (< 0.5), Q2 (0.5 ~ 0.667), Q3 (1.5 ~ 2.0), and Q5 (> 2.0) (NC-vs-SENP5 KD). **E** Heat map of the amino acid compositions of the lysine SUMOylation sites showing the frequency of different types of amino acids around SUMOylation lysine. **F** the top 3 strikingly SUMOylation motifs and conservation of SUMOylation sites are shown (motif score > 15.0).

# Figure S6

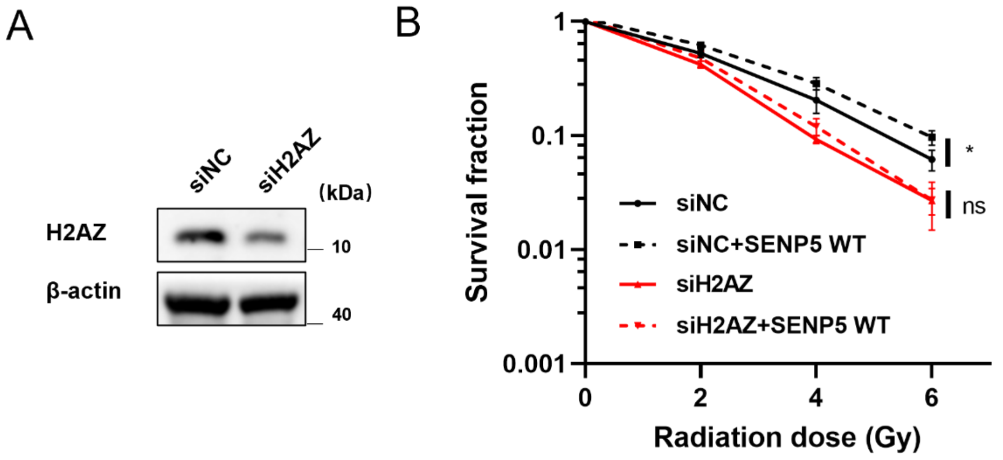


**Figure S6 SUMOylation of H2AZ play critical role in SENP5 mediated HR repair.**

**A** Knockdown of H2AZ with H2AZ-siRNA in HCT116 cells. **B** colony formation analysis of with H2AZ knockdown HCT116 cells rescued with wild type SENP5 after 0, 2, 4 and 6Gy irradiation. *P<0.01 Vs NC transfected cells. Ns Vs siH2AZ transfected cells.

# Figure S7

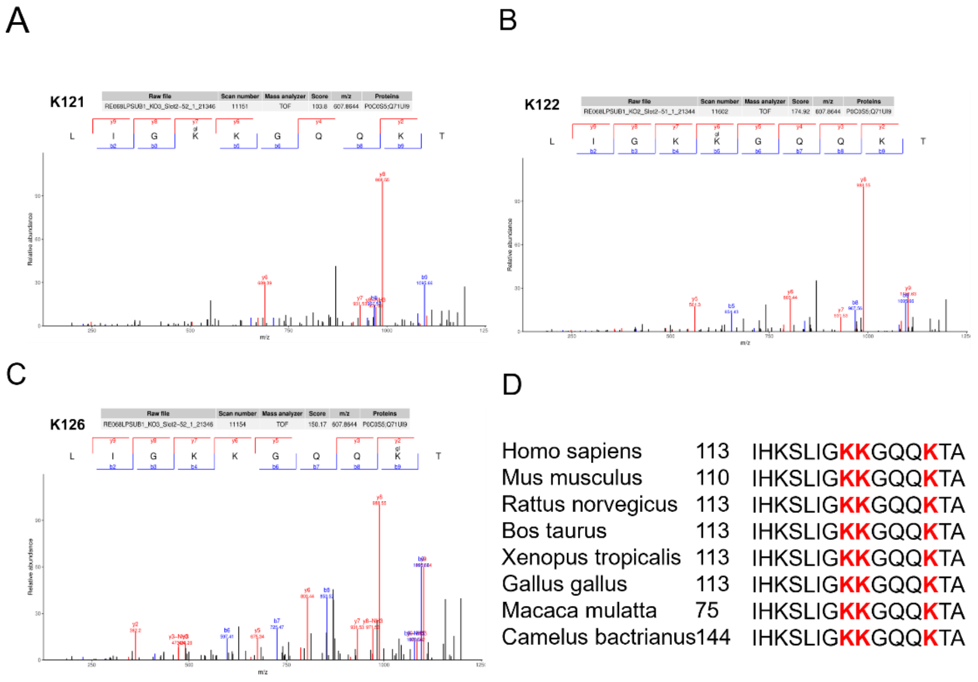


**Figure S7 SUMOylation of H2AZ play critical role in SENP5 mediated HR repair.**

**A-C** H2AZ was SUMOylation at K121 (A), K122 (B) and K126 (C). **D** The sequence of

the region containing the SUMOylation site of H2AZ was aligned with those from various species.
