# Supplementary material for: SENP5 promotes homologous recombination-mediated DNA damage repair in colorectal cancer cells through H2AZ deSUMOylation
Source: J Exp Clin Cancer Res. 2023 Sep 8;42:234. doi: 10.1186/s13046-023-02789-9 (PMC10486113; doi:10.1186/s13046-023-02789-9)
Supplement: Supplementary file 4 — Supplementary Material 4 [file 13046_2023_2789_MOESM4_ESM.docx]

**SUMO mass spectrometry**

***Cell samples and protein extractions***

To investigate the role of SENP5 through proteomics, we used irradiated NC and SENP5 KD cell proteins for SUMO modification analysis. Protein sample was sonicated three times on ice using a high intensity ultrasonic processor in lysis buffer (50μM PR-619, 1% protease inhibitor cocktail). The remaining debris was removed by centrifugation at 12,000 g at 4 °C for 10 min. Finally, the supernatant was collected and the protein concentration was determined with BCA kit according to the manufacturer’s instructions.

***Trypsin digestion***

The protein suspension was added with pre-cooled acetone and precipitated at -20℃ for 2 h. 4500 g, centrifuge for 5 min, discard the supernatant, wash and precipitate with precooled acetone twice. The precipitation was dried and 200 mM of TEAB was added for re-suspension. Then, WaLP was added at 1:50 WaLP-to-protein mass ratio for digestion overnight. The protein solution was reduced with 5 mM dithiothreitol for 30 min at 56 °C and alkylated with 11 mM iodoacetamide for 15 min at room temperature in darkness.

***Affinity Enrichment***

To enrich modified peptides, tryptic peptides dissolved in NETN buffer (100 mM NaCl, 1 mM EDTA, 50 mM Tris-HCl, 0.5% NP-40, pH 8.0) were incubated with pre-washed antibody beads (PTM1104, PTM Bio, China) at 4°C overnight with gentle shaking. Then the beads were washed for four times with NETN buffer and twice with H2O. The bound peptides were eluted from the beads with 0.1% trifluoroacetic acid. Finally, the eluted fractions were combined and vacuum-dried. The drained peptides were desalted according to the C18 ZipTips instructions (Millipore) before being used for liquid chromatography-mass spectrometry analysis.

***4D Mass Spectrometer***

The peptides were dissolved in solvent A (0.1% formic acid and 2% acetonitrile) and separated using NanoElute ultra-high performance liquid phase system. The tryptic peptides were dissolved in solvent A (0.1% formic acid, 2% acetonitrile/in water), directly loaded onto a home-made reversed-phase analytical column. Peptides were separated with a gradient from 6% to 24% solvent B (0.1% formic acid in acetonitrile) over 40 min, 24% to 32% in 12 min and climbing to 80% in 4 min then holding at 80% for the last 3 min, all at a constant flow rate of 450 nL/min on a nanoElute UHPLC system (Bruker Daltonics). The peptides were subjected to capillary source followed by the timsTOF Pro (Bruker Daltonics) mass spectrometry. The electrospray voltage applied was 1.60 kV. Precursors and fragments were analyzed at the TOF detector, with a MS/MS scan range from 100 to 1700 m/z. The timsTOF Pro was operated in parallel accumulation serial fragmentation (PASEF) mode. Precursors with charge states 0 to 5 were selected for fragmentation, and 10 PASEF-MS/MS scans were acquired per cycle. The dynamic exclusion was set to 24 s.

***Database search***

The resulting MS/MS data were processed using MaxQuant search engine (v.1.6.15.0). Tandem mass spectra were searched against the human NCBI database (Homo_sapiens_9606_SP_20220107) concatenated with reverse decoy database. Trypsin/P was specified as cleavage enzyme allowing up to 5 missing cleavages. The mass tolerance for precursor ions was set as 20 ppm in first search and 5 ppm in main search, and the mass tolerance for fragment ions was set as 0.02 Da. Carbamidomethyl on Cys was specified as fixed modification, and sumoylation on Lys were specified as variable modifications. FDR was adjusted to < 1%.

***Functional enrichment and clustering analysis***

Gene Ontology (GO) annotation was performed using the UniProt-GOA database (www. http://www.ebi.ac.uk/GOA/). The sumoylation proteins were then further classified by GO annotation based on three categories: biological process, cellular component and molecular function. The differentially expressed modified proteins in the comparison group were further divided into four parts, called Q1 to Q4 : Q1 (0 < ratio < 0.5), Q2 (0.5 < ratio < 0.667), Q3 (1.5 < ratio <2.0), and Q4 (ratio >2.0）, and further performed functional enrichment clustering analysis. Firstly, we collated all the categories that were obtained after enrichment along with their P values, and then filtered for those categories which were at least enriched in one of the clusters with P value <0.05. This filtered P value matrix was transformed by the function x =−log10 (P value). Finally, these x values were z-transformed for each functional category. These z scores were then clustered by one-way hierarchical clustering (Euclidean distance, average linkage clustering) in Genesis. Cluster membership were visualized by a heat map using the “heatmap.2” function from the “gplots” R-package v.2.0.3 (https://cran.r-project.org/web/packages/cluster/).

***Subcellular localization analysis***

The cells of eukaryotic organisms are elaborately subdivided into functionally distinct membrane bound compartments. Some major constituents of eukaryotic cells are: extracellular space, cytoplasm, nucleus, mitochondria, Golgi apparatus, endoplasmic reticulum (ER), peroxisome, vacuoles, cytoskeleton, nucleoplasm, nucleolus, nuclear matrix and ribosomes. Wolfpsort is an updated version of PSORT/PSORT II for the prediction of eukaryotic sequences.

***Motif analysis***

We used the MoMo analysis tool, based on the motif-x algorithm, to analyse the motif characteristics of the sumoylation protein sites. Peptide sequences consisting of 10 amino acids upstream and downstream of all identified modification sites were used as the target of analysis; the background of analysis was peptide sequences consisting of 10 amino acids upstream and downstream of all potentially occurring modification sites in the species. When the number of peptides in a characteristic sequence form is greater than 20 and the p-value <0.000001, the characteristic sequence form is considered to be a motif of the modified peptide. Based on the results of the MoMo analysis, a heat map was used to show the scoring of the degree of change in the frequency of amino acid occurrence near the modification site (DS). The DS was calculated as follows. DS = -Log10(p.value)∗sign (diff.percent)
