# Supplementary material for: SENP5 promotes homologous recombination-mediated DNA damage repair in colorectal cancer cells through H2AZ deSUMOylation
Source: J Exp Clin Cancer Res. 2023 Sep 8;42:234. doi: 10.1186/s13046-023-02789-9 (PMC10486113; doi:10.1186/s13046-023-02789-9)
Supplement: Supplementary file 1 — Supplementary Material 1 [file 13046_2023_2789_MOESM1_ESM.pdf]

海军军医大学
NAVAL MEDICAL UNIVERSITY

Yanyong Yang, Ph.D.
Department of Radiation Medicine
Naval Medical University
(The Second Military Medical University)
800 Xiangyin Rd., Shanghai 200433, China
Tel: +86-21-81871148
Email: yyyang2010@163.com

July 22, 2023
Prof. Mauro Castelli, Ph.D.
Regina Elena National Cancer Institute, Italy
Email: editorialjeccr@gmail.com.

Dear Prof. Mauro Castelli,

Thank you very much for your help and consideration, as well as the encouraging comments from the review concerning our manuscript entitled “SENP5 deSUMOylates H2AZ to promote homologous recombination-mediated DNA damage repair in colorectal cancer cells” (Manuscript Number: JECC-D-23-00309). These comments are all valuable and very helpful for revising and improving our paper, as well as providing important guidance to our research. According to the suggestions, we have reorganized the data, performed new experiments (Including Figure S3 A-H, S6 A-B), and corrected the improper interpretations. In addition, we have invited a native English professor to help polish our article. We hope all these changes will greatly improve the manuscript and meet the standard of your prestigious Journal of Experimental & Clinical Cancer Research. We have highlighted all the revisions in red text in the manuscript. Our point-to-point responses to the queries raised by the reviewers are listed below. We hope that these revisions successfully address their concerns and requirements and that this manuscript will be accepted. Looking forward to hearing from you soon.

With best regards,
Yanyong Yang, PhD,
Associate professor
Department of Radiation Medicine
Naval Medical University

Reviewer reports:

Reviewer #1: In this manuscript, the authors identified SENP5 (Sentrin/SUMO-specific protease 5), a member of the SENP family of SUMO (Small Ubiquitin-like Modifier)-specific proteases, as a potent radioresistant gene in colorectal cancer (CRC).
The identification of radioresistant genes can provide novel targets for combined treatments and prognostic markers by helping to better understand the molecular mechanisms underlying radiation resistance and guiding the development of more effective treatments.
Radiotherapy resistance occurs when cancer cells develop mechanisms to evade the cell-killing effects of radiation therapy. One way this can happen is through the activation of DNA damage response pathways that allow cells to repair the damage caused by radiation.
Several studies have suggested that SUMOylation can play a role in radiotherapy resistance by regulating the DNA damage response. In particular, SENP5 has been implicated in this process. However, the exact role of SENP5 in radiotherapy resistance is still being investigated, but it appears to be involved in regulating the DNA damage response through the modulation of SUMOylation.
In this manuscript, the authors correlated the expression of SENP5 with poor prognosis and radio resistance. Moreover, they characterized H2AZ as a deSUMOylation substrate of SENP5. They demonstrated that SENP5 promotes homologous recombination through deSUMOylation of H2AZ. The authors showed that SENP5 could interact with H2AZ, and that the knockdown of SENP5 led to an increase in H2AZ SUMOylation levels in cells.
Using PDO and PDX, they found that SENP5 knockdown combined with IR inhibits tumor growth. It is possible that other deSUMOylation enzymes or DNA repair pathways may compensate for the loss of SENP5 function in certain contexts, and the extent to which SENP5 regulates HR-mediated DDR in different cell types and under different conditions requires further investigation.

Reply: We gratefully thank the Reviewer #1 for his/her time spending to make the constructive remarks and useful suggestions, which has significantly raised the quality of the manuscript and has enable us to improve the manuscript. Each suggested revision and comment, brought forward by the Reviewer #1 was accurately incorporated and considered. All the suggestions were addressed and responded point by point below, and all the revisions are also indicated in the main text.

Major comments:
1.In Fig S2A, the authors showed the efficiency of shRNA against SENP5, but the silencing is not complete. Authors should test the effect of the different sh-SENP5 sequences on proliferation, apoptosis, and cell cycle to test if the different amount of

protein is still able to induce the inhibition of cell growth. Moreover, the authors should state which of the sequence has been used for the experiments.

8. The efficiency of shRNA on protein levels is low, the amount of residual protein (almost 50%) could create an indirect effect.

Reply: Thank you for your valuable comments. Since the first and eighth questions are mainly related to lentiviral knockdown of SENP5 protein expression levels, we will reply to the above questions together. As you suggested, the lentiviral approach did not completely deplete SENP5 protein, but it also had a significant knockdown efficacy (The sh2 sequence knocked down SENP5 protein expression by 70% in HCT116 cells and by 60% in HT29 cells). Therefore, we finally chose the lentiviral mediated SENP5 knockdown to investigate its role in DNA damage repair and cancer resistance.

In our experimental system, both shSENP5-1 and shSENP5-2 sequences had a significant knockdown effect on SENP5. Among them, the knockdown efficacy of shSENP5-1 was about 50%, while shRNA-2 inhibited almost 60-70% of SENP5 protein expression. Therefore, in the initial manuscript, we used shSENP5-2 sequences to knockdown SENP5 in the experiments including CCK8 assay, clone formation assay, apoptosis assay and cell cycle assay (Fig. 2D-H, S2B-K). Following your suggestion, we also used SENP5 knockdown cells constructed with shSENP5-1 sequences to perform the following experiments, including cell proliferation, cell apoptosis and cell cycle. The results showed that knockdown of SENP5 using shSENP5-1 also significantly inhibited cell proliferation, promoted cell apoptosis, and delayed cell cycle progression in tumor cells after irradiation (Fig. S3). CCK8 assay showed that HCT116 and HT29 cells constructed by shSENP5-1 showed significant inhibition of cell proliferation after irradiation (Fig. S3A-D). Flow cytometry apoptosis assay further showed that the apoptosis rate of shSENP5-1 cells increased significantly after irradiation (Fig. S3E-F). The results of cell cycle assay suggested that knockdown of SENP5 also significantly aggravated cell cycle arrest (Fig. S3G-H). In summary, the results of cells constructed by shSENP5-1 and shSENP5-2 both suggested that knockdown of SENP5 significantly inhibited the growth of colorectal cancer cells.

In response to question 8, although shRNA does not completely deplete SENP5 protein expression, the lentiviral sequence of shRNA1 and shRNA2 can significantly inhibit tumor proliferation in both HCT116 and HT29 cells, which provided essential evidence for that SENP5 is an effective target for radioresistance.

2.In Fig 4G, the amount of SENP5 after silencing is not shown. In a number of experiments (S3F, S3G), the WB of the silenced protein is not shown. This should have been an important control in such experiments.

Reply: We greatly appreciate your suggestions. As you suggested, the western blotting images of the silenced protein should be a very important result. In fact, we have

detected the expression of SENP5 when performing the western blotting experiments. All the experiments were conducted after confirming the knockdown efficacy of SENP5. To add rigor to our study, we supplemented the bands for SENP5 in the revised version (Fig. 4G, S3F, S3G). Thanks again for the reminder.

3.Fig. 6 is not clearly described. Cells have been irradiated but there is no indication about dose or timing. The IR labels in Fig. 6 are confusing.
In Fig. 2A the authors showed an increase in the SENP5 levels after irradiation. In IP experiments, the expression levels of SENP5 after IR in the INPUT are not increased, in HCT116 cell lines it looks even less compared to not irradiated cells.

Reply: Thanks for the kind suggestions. The irradiation doses used for the immunoprecipitation experiments in figure 6 are all 8 Gy. Then cells were collected at 8 h after irradiation. We have supplemented the irradiation dose and timing in the methods and figures in the revised manuscript. In addition, we have further revised the labeling in the figures to indicate which group was irradiated or not.
Regarding the SENP5 protein expression after irradiation, we repeated the experiment several times and found that the expression of SENP5 protein increased with time after irradiation. This can also be found from the results of SENP5 protein in HCT116 and HT29 cells (Fig. 2A).
Regarding your mention that the expression of SENP5 protein was not significantly upregulated after irradiation in the IP experiment, this is mainly because in the IP experiment, we optimized the SENP5 expression to equal level based on raw density to compare the differences in binding efficacy with H2AZ. Taking figure 6A as an example, we took the protein SENP5 recruited by the magnetic beads as a control, and then focused on the amount of H2AZ protein bound to SENP5 after irradiation.
In addition, the method is often used to quantify the results of IP experiments (*for instance, Yu X, J Hematol Oncol. 2020, PMID: 33101770; Liu W, Adv Sci (Weinh). 2023, PMID: 36563124; Zeng X, Nat Cancer. 2022, PMID: 36138131)*. We also add a description to the method of IP experiments and reference to avoid misunderstandings.

4.Figures S3F and S3G are misplaced, the authors described in the main text, line 13, page 15, "the number of 53BP1 foci was normal (Fig. 3J, 3K; S3F, S3G)". Fig S3F and S3G represent instead Western blot analysis. They should correct the label of the Figure in the text with S3D, S3E.
5.As before, in line 20, page 15, the authors describe the effect of the knockdown of SENP5 on the phosphorylation of key HR repair proteins (Fig. S3D, S3E). The results correspond instead to Fig. S3F and S3G.

Reply: Thank you for your careful reading of our manuscript. We apologize for the confusion and appreciate the valuable suggestions. Since the fourth and fifth questions

refer to the similar mistakes in the manuscript, we will answer them together. In the revised version, due to the addition of Figure S3 in the results, the name of Figure S3 in the first version was adjusted to Figure S4, that is, Figures S3D-G was renamed Figures S4D-G. Figures S4D and S4E correspond to immunofluorescence and statistical plots of 53BP1 foci. Figure S4F and S4G are the effects of knocking down SENP5 on phosphorylation of key HR repair proteins in HCT116 and HT29, respectively. We have accurately matched the results to the figures in the revised version. For question 4, 53BP1 foci results correspond to S4D and S4E. In responsive to question 5, the results of the effect of knockdown of SENP5 on phosphorylation of key HR repair proteins correspond to S3F and S3G. Thank you so much for your careful check, and the label mistake has been corrected in the revised manuscript. We feel sorry for our carelessness.

6.Indication about MW is missing in almost all the Figures, except for Fig. S3F, S3G.

Reply：We are very grateful for the suggestions you made. For the consistency and rigor of the article, we have added the MW of markers in the WB bands of all Western blot assay. Including Fig. S1D; Fig. 2A, S2A; Fig. S3F-G; Fig. 4G; Fig. 6A-G; Fig. 7B-C. Thank you again for your valuable advice.

7. Indication about the Gy used should be included in the Figure and in the main text, not only in the Figure legend, as such authors did for Fig. 2B, 3D.
In a number of experiments, the indication of the dose of irradiation is not stated, and it is not constant. In fact, some experiments have been performed with a range of doses (0-2-4-6Gy), some experiments with 5Gy, and others with 8Gy. Could the authors explain the reason behind this continuous change?

Reply：Thank you for your constructive suggestions. For specific purpose of experiments, we used different irradiation doses for different experiments. Based on the suggestions, we have labeled the irradiation doses directly in all the Figures, and the rest are illustrated in the figure legend. In general, to test the radiosensitivity of the cells, different radiation doses (2/4/6Gy) were applied to detect the clonogenic ability of the cells as previously described (*Wang Y, Nat Commun. 2019, PMID: 6391399; Bennett L, Nucleic Acids Res. 2020, PMID:31799632*). Based on the results of colony formation assay, we found that knockdown of SENP5 significantly inhibited the cell proliferation after irradiation. To investigate the specific mechanism of SENP5 in regulating radiosensitivity, we explored the DNA damage repair mechanism by immunofluorescence, neutral comet assay, WB and IP experiments. We performed many pre-experiments to explore the number of cell foci at irradiation doses of 2, 3, 4, 5, and 6 Gy and found that cells undergoing irradiation doses exceeding 5 Gy showed overlapping foci that could not be easily counted. In the above experiments,

immunofluorescence experiments generally utilized 5 Gy irradiation to detect clearly defined foci. In addition, based on our studies and optimization, the cells were often irradiated with 8 Gy for the neutral comet experiment, WB and IP experiments (*Lei X, J Exp Clin Cancer Res.2021, PMID: 34225780*). Therefore, different radiation doses were used in different experiments in this article. Thank you for your kind suggestion. We also annotate it in the figure legend to better show the results.

Minor comments

1.I am sure that this would be taken care of in the copy-editing stage, but there are a number of sentences that are not very good English. Please go through the manuscript and make the sentences as clear as possible.

Reply：Thank you very much for your suggestion. We read through the manuscript carefully and revised some sentences in the article that did not make sense. We also had the language reviewed by a native English-speaking professor to revise the language. Thank you again for your advice.

2. The authors made large use of "etc", in the abstract, in the background, and in the results section. Instead of writing etc., explicitly state the words or list that you are alluding to with your use of etc.

Reply：Thank you for your suggestions. We have made the following modifications: 1. In the abstract, in the summary of experimental methods, we have listed all the experiments addressed in the article, including CCK8, clone formation, comet assay, immunofluorescence and flow cytometry analysis of apoptosis and cell cycle. 2. In the background, we added that DSB can trigger a series of cellular DNA damage responses (DDRs) that lead to a variety of cellular responses such as cell cycle arrest, apoptosis, autophagy and senescence. 3. In the results, we have selected the most important DNA repair pathways affected by downregulation of SENP5 are described, supplemented with mismatch repair and base excision repair. We have carefully checked the full text to add the content of the abbreviations represented by etc.

3. Line 27, page 2, PDO and PDX models WERE used...
4. Line 42, page 3, "...which is an important upstreaming molecular event THAT determines cancer cell apoptosis...".
5. Line 13, page 9, "at a concentration of 2x105 cells/mL".
6. Line 36, page 10, "Four-week-old athymic nude mouse strain WERE used...".
9. Line 51, page 15, there is a typo: clonogenic assay instead of colonogeic.
10. Line 53, page 15, "...increased celluLar resistance...".
11. Line 17, page 17, SUMOylation of H2AZ playS a critical role in SENP5-mediated HR repair.

14. Line 31, page 20, "H2AZ IS a direct deSUMOylation substrate of SENP5".
17. Figure legend S3 F-G, knockdown is misspelled.

Reply：We really appreciate your suggestions. Questions 3-6, 9-11, 14 and 17 are all grammatical problems and typos in the article, and we sincerely apologize for any inconvenience caused by the above problems to the reading. We have seriously revised the above questions. Next, we listed the initial and revised sentences one by one, with the changes highlighted in red.

For question 3, we have corrected the “*First of all, PDO and PDX models was established by using the tumor tissues surgically resected from rectal cancer patients in Changhai hospital (Fig. 8A)*” into “First of all, PDO and PDX models were established by using the tumor tissues surgically resected from rectal cancer patients in Changhai hospital (Fig. 8A)”.

For question 4, we have amended the original article from “*The most severe type of DNA damage induced by ionizing radiation (IR) is double strand breaks (DSBs), which is an important upstreaming molecular events determines cancer cell apoptosis, autophagy, senescence, cell cycle arrest etc*” to “*The most severe type of DNA damage induced by ionizing radiation (IR) is double strand breaks (DSBs), which is an important upstreaming molecular events that can trigger a series of cellular DNA damage responses (DDRs) and lead to a variety of cellular responses such as cell cycle arrest, apoptosis, autophagy, and senescence*”.

For question 5, we have corrected the “*at a concentration of 2 × 105 cells/mL*” into “*at a concentration of* 2×10^5 *cells/mL*”.

For question 6, we have corrected the “*Four-week-old athymic nude mouse strain were used for the xenograft tumor assay in this study.*” into “*Four-week-old athymic nude mouse strain was used for the xenograft tumor assay in this study*”.

For question 9 and 10，we correctly revised “*In colonegeic assay, re-expression of SENP5 wt, instead of SENP5 C713L mutant, significantly increased celluLar resistance to IR (Fig. 4D)*” to “*In clonogenic assay, re-expression of SENP5 wt, instead of SENP5 C713L mutant, significantly increased cellular resistance to IR (Fig. 4D)*”.

For question 11，we have corrected the “*SUMOylation of H2AZ play critical role in SENP5 mediated HR repair*” into “*SUMOylation of H2AZ plays critical role in SENP5 mediated HR repair*”.

For question 14，we have corrected the “*Further data revealed that H2AZ as a direct deSUMOylation substrate of SENP5*” into “*Further data revealed that H2AZ is a direct deSUMOylation substrate of SENP5*”.

For question 17, in figure legend S3 F-G, we have corrected the word “*konckdown*” to “*knockdown*”.

In addition, the entire text was carefully checked to avoid similar problems. Thank you again for your valuable comments, which are crucial to improving the quality of our article.

7. Line 15, page 13, "S1C results for other CELL LINES were not shown". Results for other genes are shown in S1B and S1C.

Reply：Thank you for your suggestions. In this paper, we only show the high-content screening results of SENP5, STK32C, PAGE2B and DMRT3 genes. Results for other genes will be presented in other subsequent articles.

8. Line 7, page 15, "we utilized a NHEJ and HR reporter assay, as previously reported". Please, include a reference.

Reply：Thank you for your valuable comments. We have supplemented the references for the NHEJ and HR experiments. (*Arnoult N, Nature. 2017, PMID: 28959974*)

12. Wild type is often misspelled along the text. For example, in Line 44, page 17.

Reply：In the manuscript, we made some mistakes such as misspelling the word wild as wide or wile. We went through the full text and changed them all to wild-type. Thank you for your valuable advice.

13. Please go through the manuscript and make the sentences as clear as possible.
- Line 60, page 19, "Often occurs in S phase, HR repair was investigated in clinical trials...". What is the relevance of the cell cycle phase?

Reply：Thank you very much for your suggestion. For the accuracy of the description, we have modified and replaced the original text. HR repair occurs mainly in the S phase and is also the main way for cells to repair damaged DNA precisely. Many basic studies have reported that inhibition of HR repair significantly inhibits the growth of tumor cells. Therefore, many clinical trials have investigated HR repair as a potential target for tumor therapy, including ATR, ATM, and Rad51 [36, 37].

- Line 44, page 20, "After possessing DSB repair...".

Reply：We are very sorry for this spelling error. The original sentence in the article is: “*After possessing DSB repair, H2AZ was removed by the nucleosome remodeler INO80 and initiate the downstream signaling of DNA repair*”. We misspelled processing as possessing. We have deleted the word possess for better understanding. In the revised version, the sentence is modified as follows: “*After DSB repair, H2AZ was removed by the nucleosome remodeling enzyme INO80 and initiated the downstream signaling of DNA repair*”.

- In the SUMO mass spectrometry section, the authors define the analysis performed for SUMO proteins as "sumo modificationomic analysis".

Reply：For clarity, we have changed the original word "sumo modificationomic analysis" into "SUMO modification analysis".

15. The conclusion paragraph could be improved. The sentences are quite redundant.

Reply：Thank you for your valuable comments. We have revised our conclusions as follows: “Our study reveals that SENP5 is a potent gene associated with radiotherapy resistance and is involved in cellular HR repair by regulating SUMO modification of H2AZ. Our findings provide a novel mechanism for radiotherapy resistance and suggest that SENP5 could be a potential target to improve radiotherapy efficacy”.

16. The funding section is identical to the ethical approval and consent to participate.

Reply：Thank you for your kind reminder. In the revised version, we have listed in detail the funds that have funded this study. We are sorry for the mistake in the first manuscript and thank you again for the reminder.

Reviewer #2: The authors report that SENP5 critically contribute to resistance of radiotherapy by controlling SUMOylation of H2A.Z histone variant for HR DNA repair. Form previous CRISPR screening, authors identified the potential role of SENP5 as a responsible gene for radiotherapy resistance, i.e. knockdown of SENP5 reduced survival of cancer cells after radiation. Clinical patients' data support that SENP5 expression level is correlated to survival rate, higher expression of SENP5 decrease the disease-free survival duration and that relationship is consistently observed after radiotherapy. Established cancer cell lines also showed consistent relationship of SENP5 expression and sensitivity to radiation. The contribution of SENP5 in HR DNA repair pathway was determine by multiple assays and gene expression analysis. These convincingly support the positive contribution of SENP5 on HR DNA repair. Authors tried to identify responsible SUMOylated protein(s) targeted by SENP5 for this phenotype and performed Mass Spectrometry analysis of SUMOylated proteins comparing irradiated cells with or without SENP5. They identified h2A.Z, histone variant known to regulate active transcription, as a potential target then examined its role on this SENP5-depndent radiotherapy resistance/HR repair.

Reply: We appreciate the Reviewer #2 for carefully reading our manuscript and making their insightful and constructive suggestions, which enabled us to prepare a greatly improved manuscript. Based on these suggestions, we have revised the statements in

our methods and conclusions to better highlight the novelty and advancement of our work.

The major issue in this study is lack of evidence that H2A.Z is SUMOylated. Mass Spectrometry was performed with enriching the potential SUMOylated peptides by the antibody that recognize diglycyllysine (PTM-1104, PTM Biolabs). By definition, the antibody cannot distinguish the Ubiquitinated protein or SUMOylated protein. In addition, the peptide was obtained by tryptic digestion which is known to retain long peptide from SUMO2/3 on modified lysine thus previous study have to use mutated SUMO2/3 for isolating by anti-diglycyllysine antibody. Does MS spectra can distinguish or specify SUMOylation? If so, I wish to see that explanation.

Reply: We apologize for our carelessness in writing WaLP as trypsin in the methods. In the revised version, we have carefully checked and revised additional file 2 and marked the modifications.
Sumo mass spectrometry sequencing in the article references the method published in Nature Communication, which effectively distinguishes between ubiquitination and SUMOylation（*Lumpkin RJ, Nat Commun. 2017, PMID: 29079793*）. We display the image from the Nature Communication for ease of illustration. As shown in Figure 1A, the C-terminal end of ubiquitin is RGG, while the C-terminal end of SUMO proteins including SUMO1, SUMO2, SUMO3, and SUMO4 is TGG. Based on the structural characteristics of sumo and ubiquitin, this study proposes a novel SUMO mass spectrometry sequencing approach using WaLP for proteolysis. WaLP is a protease with a specific cleavage propensity that has a very high propensity to cleave the C-terminus of four amino acids, A, T, S and V, but has almost zero relative cleavage capacity for the basic amino acids H, K and R. This is a very important feature for the enrichment of SUMOylated protein, because only if this protease has a selective propensity to cleave the C-terminus of threonine rather than the C-terminus of arginine, the peptide with KGG residues we enriched will be from SUMO modification rather than ubiquitinated modification. Furthermore, in addition to SUMO sequencing suggesting SUMOylation of H2AZ, we also validated in cellular experiment (Fig. 6G).

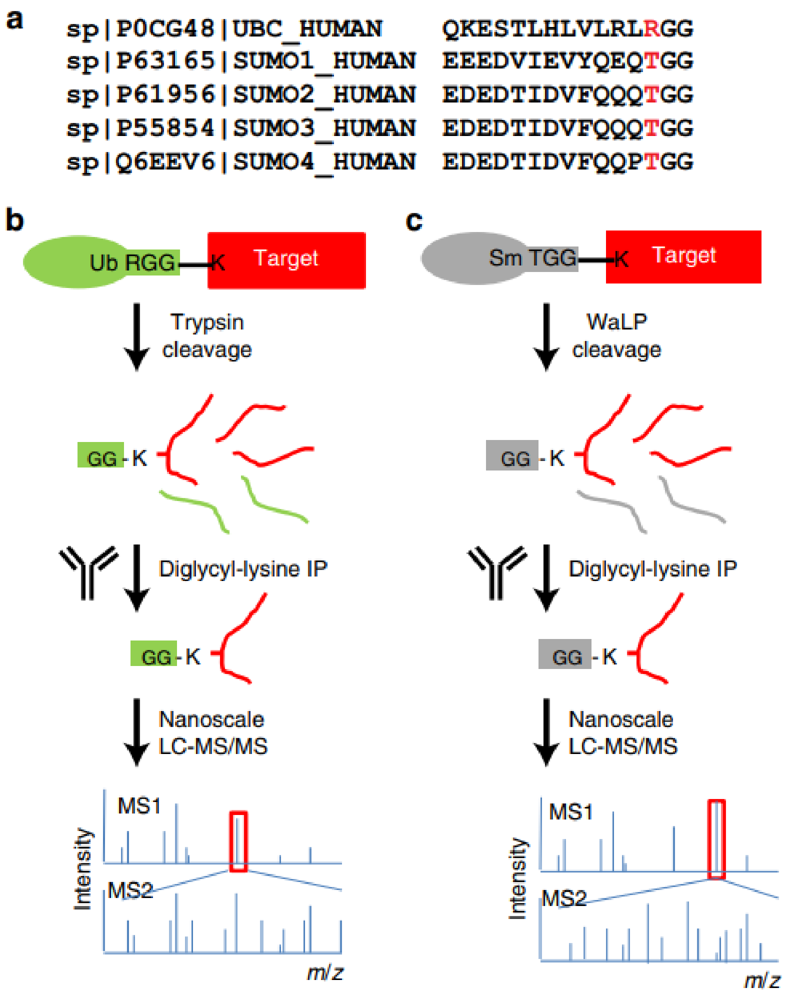

Figure 1（*Nature communication, 2017, PMID: 29079793*）
A strategy for mapping endogenous SUMO-modification sites.

Figure 6 and Figure 7 lack information of molecular weight of H2A.Z band thus unable to interpret if the band is SUMOylated H2A.Z. I could not understand why co-immunoprecipitation can be the evidence of SUMOylation of H2A.Z. Why can SUMOylated substrate form stable complex with highly active deSUMOylation enzyme?

Reply: Thank you so much for your valuable suggestions. In the revised version of Figure 6 and Figure 7, we have added markers for molecular weight for all bands in western blotting assay. We also checked all the western blot images and added markers for molecular weight in other Figures to make it consistent and precise.
Indeed, the co-immunoprecipitation could not directly show the SUMOylation of H2AZ. However, we do observe a shift of molecular weight of the immunoblotted band of H2AZ, the increase of MW may indicate the modification of H2AZ proteins. To validate the SUMOylation of H2AZ, we performed an immunoprecipitation with H2AZ specific

antibody, after which SUMO2/3 was detected with immunoblotting. To be expected, the SUMOylation of H2AZ was detected in irradiated cells, and was upregulated in SENP5 knockdown cells (Figure 6G). These data showed that SUMOylation of H2AZ was related to radiotherapy and was possibly regulated by SENP5.

Similar to other post-translational modifications like phosphorylation, ubiquitination, the SUMOylation and deSUMOylation balance of protein was regulated in a dynamic process in response to multiple stimuli. In our model, the SUMOylation of H2AZ was observed after irradiation and was then deSUMOylated by SENP5. Thus, the SUMOylated H2AZ did not form stable complex with SENP5, but interact with SENP5 in a dynamic state. So, there is some amount of SENP5 interact with H2AZ during the deSUMOylating process. This state makes it possible to be detected in a specific time window when the deSUMOylation process exist. In our experiments, we performed co-immunoprecipitation at8 h after irradiation and detected the interacting of SENP5 with SUMOylated H2AZ in HCT116 cells.

If the immunoprecipitation is done with mutant SENP5 that lacks deSUMOylation activity, it might be possible and we should see SUMOylated H2A.Z which increased molecular weight. If the mutation of lysine on H2A.Z abrogating the interaction because of lack of SUMOylation, the band precipitated with SENP5 should be the SUMOylated H2A.Z, thus molecular weight should be increased.

Reply: Thank you very much for your suggestion. Indeed, the SUMOylation of H2AZ should be validated in multiple assays. Through both endogenous and exogenous cellular experiments, we have observed SUMOylated H2AZ protein binding with SENP5 (Fig 6A-F). As for the immunoprecipitation with mutant SENP5 lacks deSUMOylation activity, it has been reported that SENP5 with the lysine mutation at site 713 (C713) did not bind to SUMO protein. This data can be found in Figure 2 (*Di Bacco A, Mol Cell Biol, 2006, PMID: 16738315*).

In our study, we identified these three lysine sites (K121, 122, 126) through Sumo mass spectrometry sequencing. SUMOylation of H2AZ was found to be abrogated when the K121, K122 and K126 were mutated. After then, we have observed increased level of SUMOylated H2AZ in SENP5 knockdown cells with increased molecular weight (Fig 6G). Indeed, the molecular weight of SUMO-modified H2AZ is increased. In addition, some lysine was often found to be potential sites for ubiquitination. In our future study, we are also in process to investigate the crosstalk between ubiquitination and SUMOylation in regulating H2AZ function.

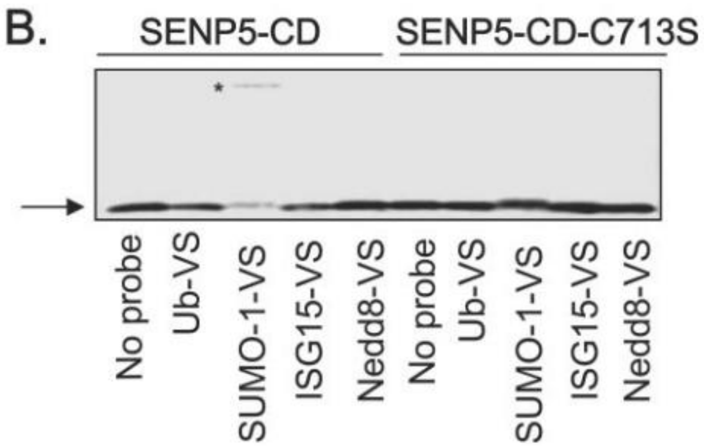

Figure 2 (*Mol Cell Biol, 2006, PMID: 16738315*)
SENP5-C713 mutant do not bind to SUMOylated proteins.

Overall, there is no evidence presented for SUMOylation of H2A.Z therefore the title of the manuscript is not justified. Because H2A.Z-3KR mutant, which SENP5 cannot interact with, phenocopy the loss of SENP5 function, it is possible that recruitment of SENP5 at H2A.Z marked loci is responsible for the phenotype. If that is the case, SENP5 might work on other SUMOylated proteins located on H2A.Z marked loci.

Overall, I have convinced that SENP5 is critically contributing to radiotherapy resistance by promoting DNA repair pathway in cancers. That information is strong and well justified by most of the assays. Because no evidence was provided to support SUMOylation of H2A.Z and its contribution of the phenotype, which is the title of the manuscript, I cannot support the publication of this manuscript as current format.

Reply: Thank you very much for the suggestions from the Reviewer #2. Based on the suggestions, we have repeated our experiments and performed new experiments (Fig. S6), which demonstrated that H2AZ play a critical role in SENP5-mediated radioresisance. In detail, we knocked down H2AZ in HCT116 cells and performed rescue experiments in H2AZ-depleted cells with SENP5 overexpression. As shown in Figure S6, overexpression of SENP5 in the NC group could significantly improve cell proliferation ability. However, SENP5 did not improve cell proliferation ability in HCT116 cells with H2AZ knockdown, indicating that H2AZ is an important molecule downstream of SENP5. These results showed that H2AZ plays a key role in SENP5 promoting radioresistance of tumor cells.
Last but not least, according to your suggestion, we have changed the title of the article from "*SENP5 deSUMOylates H2AZ to promotes homologous recombination-mediated DNA damage repair in colorectal cancer cells*" to "*SENP5 promotes homologous recombination-mediated DNA damage repair in colorectal cancer cells through H2AZ deSUMOylation*".

Reviewer #3: The manuscript described a novel role of SENP5 responsible for radiotherapy resistance in colorectal cancer (CRC). Through high-content screening and tissue array analyses, Liu and his colleagues found that SENP5 may be related to radioresistant in CRC patients. Silenced expression of SENP5 could significantly increase the radiosensitivity in CRC cells. Detailed analysis revealed that SENP5 could promote HR-mediated DNA damage repair through deSUMOylation of H2AZ, which affects its DNA binding efficacy and the recruitment of the downstream HR factors. Thus, SENP5 could serve as a potential target to improve the effectiveness of radiotherapy in CRC. Overall, it is a fascinating paper with logical thinking and qualified results. All the findings provided in the manuscript are consistent and strongly support their crucial conclusion. What they discovered in this study is not only a novel mechanism of radioresistant but also provides a novel theoretical basis for clinics to overcome resistance to radiotherapy in CRC patients in the future. Only a few minor points need to be modified that can help to improve the exciting findings in the manuscript.

Reply: We appreciate the reviewer's insightful and constructive comments and advice, and we have carefully addressed these concerns and made a proper revision of the manuscript. These comments and suggestions have not only enabled us to provide a highly improved manuscript but also inspired us to conduct more in-depth studies on the role of SENP5 in the regulation of tumor radiosensitivity in future works. All the suggestions were addressed and responded point by point below, and all the revisions are also indicated in the main text.

1.In Figure 7, the authors showed that H2AZ is the direct target of SENP5, and its' SUMOylated status was critical for SENP5-mediated HR repair in vitro. Strongly suggest the authors can try to detect the expression patterns of SUMOylate H2AZ in the tissues of PDX mice in Figure 8. This will be more helpful in convincing readers about their critical findings in this study.

Reply: We gratefully appreciate for your valuable suggestion. In the tumor tissues isolated from the PDX, we tried to detect SUMO-modified H2AZ by immunohistochemical methods. However, there is no commercially available H2AZ antibodies that specifically recognize SUMOylated H2AZ. At present, we are also trying to customize the detection of SUMOylate antibodies Unfortunately, for some technical reasons, we cannot obtain this SUMO-specific H2AZ antibody at this time. In addition, we also tried to detect the SUMO modification of H2AZ by PDX tumor samples, and the results are shown in Figure 1. We increased the loading volume of the sample, and the protein bands modified by SUMO were vaguely visible. This may be due to the fact that PDX was collected about 2 weeks after irradiation, but in cell experiments, proteins

were collected 8 hours after irradiation. The SUMOylation of the protein is not easily detectable due to the long time after irradiation. We consider this result unrepresentative and therefore do not recommend displaying it in the article. Thank you again for your valuable advice.

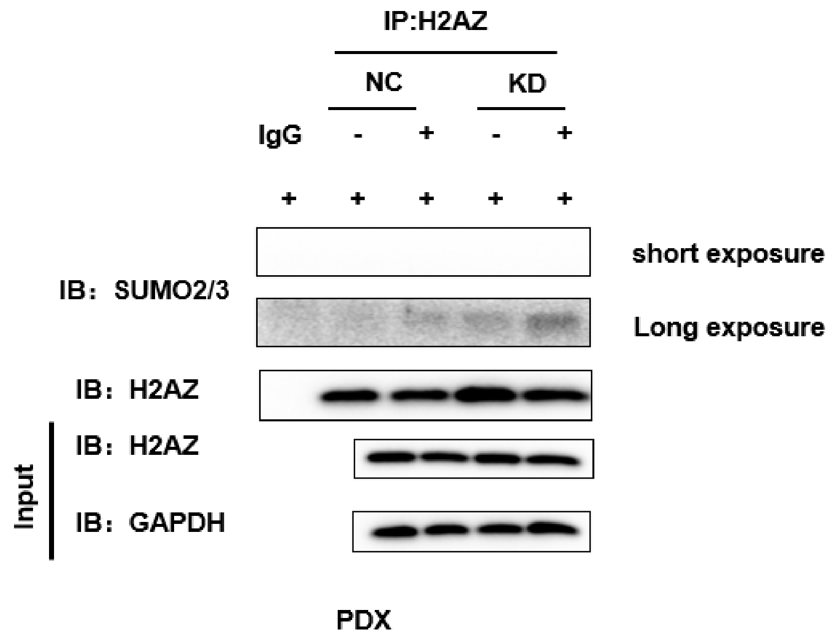

Figure1: SUMO2/3 were detected in PDX mice with protein immunoprecipitated with H2AZ antibody.

2.Some of the changes in the immunoblotting of Figures 6A, B, C, D, and G could be more dramatic; it would be best if the authors could provide the quantified results of the immunoblotting.

Reply: Thank you very much for your valuable advice. We quantified the key data in plots 6A, B, C, D, and G using image J and supplemented the quantification results below the corresponding bands.

3.The English editing should be checked in more detail. Some typing errors need to be corrected.

(1) on page 23, line 16: the sentence "he Ethics Committee of Naval Medical University" is duplicated and should be deleted.

(2) on page 23, the description of Funding seems more focused on the proof of their experimental procedures rather than on the source of their funding. It should be corrected.

(3) some of the authors' contributions are repeated. For example, "T.L., H.W., Y.C.,

Y.Y., and Z.W. study concept and design, carried out experiments, preparation of the manuscript, obtain funding" and " J.C., W.Z., F.G., Y.Y. study design, obtained funding." Please check it in more detail.

(4) The legend of Figure 1A should be modified as "Expression of SENP5 in CRC patients in colorectal cancer tissues and normal tissues. These data were acquired from UALCAN (http://ualcan.path.uab.edu)".

Reply: Thanks for your kindly reminder. We are really sorry for these mistakes. We have revised the above questions one-to-one and highlighted them in the revised manuscripts. In response to question (1), we have removed duplicate statements and carefully checked the full text again to avoid similar situations. For question (2), we have added the names and numbers of the fund that had financial support to this research. In response to question (3), we carefully examined the workload of the authors in this article. In the revised version, we have provided a detailed description of each author's contribution to this paper to better highlight the value of the author's contribution. In response to question (4), we revised the legend of Figure 1 in the main text. In addition, we have also checked the whole manuscript to avoid spelling and editing mistakes. All the improper expressions were also corrected. Thank you again for your careful consideration, which has greatly helped to improve the quality of our manuscript.

We are looking forward to hearing from you for your decision. If you have any additional queries, please do not hesitate to contact us.

With best regards,

Yanyong Yang, PhD,
Associate professor
Department of Radiation Medicine
Naval Medical University
